# The Versatility of Diazirines: Properties, Synthetic and Modern Applications

**DOI:** 10.1002/chem.202500414

**Published:** 2025-07-11

**Authors:** Mathieu L. Lepage, Emmanuel Gras

**Affiliations:** ^1^ Laboratoire Hétérochimie Fondamentale et Appliquée (UMR 5069) CNRS / Université de Toulouse 118 route de Narbonne 31062 Toulouse France

**Keywords:** carbenes, diazirines, organic synthesis, photochemistry, reactivity

## Abstract

Diazirines are 3‐membered heterocycles containing two nitrogen atoms connected by a double bond. They are mostly known for their usage in photoaffinity labeling (PAL), due to their stability and their facile photolysis for on‐demand carbene generation. Yet diazirines possess a multi‐faceted reactivity that also holds great potential for organic synthesis. This is illustrated in the present review, which is meant to be a beginner's guide for new diazirine users. After briefly summarizing the main synthetic approaches to these derivatives (with a focus on recent improvements), we emphasize some of their most critical features and properties before describing the various modes of activation toward carbene generation, some of which were only uncovered in the past decade. We then review the many modern uses of diazirines, underlining their underappreciated versatility: as carbene precursors in synthesis, but also as electrophilic nitrogen donors, as NMR hyperpolarization probes, or as molecular tools in materials science.

## Graphical abstract

Diazirines are 3‐membered heterocycles containing two nitrogen atoms connected by a double bond. They possess a multi‐faceted reactivity that holds great potential for organic synthesis. This review discusses their properties, modes of activation, and their many modern uses, underlining their underappreciated versatility: as carbene precursors, but also as electrophilic nitrogen donors, as NMR hyperpolarization probes, or as molecular tools in materials science.

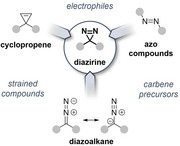

## 1 Introduction

**Figure 1:**
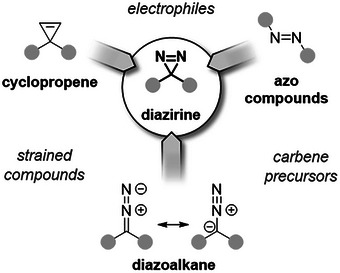
Diazirines combine features and properties of several structurally related motifs.

Diazirines are three‐membered heterocycles containing two nitrogen atoms connected by a double bond. They are the cyclic structural isomers of diazoalkanes and combine structural features of cyclopropenes and azo compounds (Figure 1). While diazo compounds have been known since the 19^th^ century, it was not until the early 1960s that diazirines were formally identified, prepared, and characterized.^[^ ^1^, ^2^, ^3^, ^4^ ^]^ Seminal works on this emerging class of compounds immediately noted their increased stability to acids,^[^ ^1^ ^]^ bases, and oxidation.^[^ ^2^ ^]^ Their propensity to form carbenes under UV irradiation was quickly established, which promoted their historical use in photoaffinity labeling (PAL).^[^ ^5^, ^6^, ^7^, ^8^ ^]^ This coincidentally growing field, where a label is covalently attached near the active site of a protein via photoactivation, drove the development of diazirines over six decades, prompting the design of new synthetic pathways and unraveling their properties. To this day, PAL remains the main usage of diazirines, and the extensive knowledge of this field is regularly and thoroughly reviewed.^[^ ^7^, ^8^, ^9^, ^10^, ^11^ ^]^

Yet there is more to diazirines than PAL: around this main body of research is gravitating a very diverse and scattered ensemble of data regarding alternative uses of diazirines. Those rely on the multifaceted reactivity of this lesser‐known class of heterocycles, inherited from their hybrid kinship with cyclopropenes and (di)azo compounds, making them not only underused carbene precursors but also radical acceptors and electrophilic nitrogen donors. This underlying knowledge has so far remained in the shadow of PAL but is growing at a fast pace as several communities come to realize their potential.^[^ ^12^, ^13^, ^14^, ^15^, ^16^ ^]^ This review is thus intended to provide a consolidated overview^[^ ^17^ ^]^ of the knowledge accumulated on diazirines beyond their usage as photochemical probes in chemical biology, along with some generalities on their properties.

## 2 Synthesis

**Figure 2:**
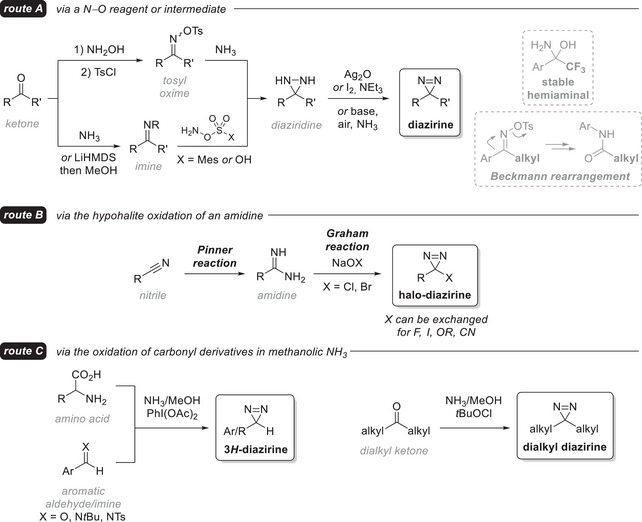
Non‐exhaustive, general overview of synthesis pathways to diazirines.

The synthesis of diazirines with various substitution patterns is regularly and comprehensively reviewed by others.^[^ ^7^, ^8^, ^9^, ^10^, ^18^, ^19^, ^20^, ^21^ ^]^ We will thus simply explain here the main synthetic pathways and highlight the most recent improvements (Figure 2). From most ketones (**route A**), access to the corresponding diaziridine is accomplished under various conditions, all involving ammonia (or a derivative) and an *O*‐sulfonyl hydroxylamine.^[^ ^8^, ^22^, ^23^ ^]^ Depending on the substituents (i.e., trifluoromethyl vs. alkyl), the order of those steps matters because aryl trifluoromethyl ketones form stable hemiaminals^[^ ^22^, ^24^, ^25^ ^]^ and because aryl alkyl tosyloximes are prone to Beckmann rearrangements. The obtained diaziridine is then readily oxidized to the diazirine with a variety of reagents, historically with silver oxide, more practically with iodine, but also in strong alkaline conditions.^[^ ^26^, ^27^ ^]^ Recently, an interesting method was developed that converts the tosyloxime intermediate directly into the diazirine by reaction with azanide NH_2_^−^, shortening the synthesis by one step.^[^ ^28^ ^]^ These sequences comprising 2–4 steps can sometimes be performed in one pot and generally with only one purification at the end. Indeed, the diazirine motif itself is extremely apolar, and derivatives bearing no other polar groups elute quickly with hydrocarbons (e.g., pentane) in regular silica gel chromatography, making the overall process straightforward and robust.

3‐Alkyl‐3‐halodiazirines (**route B**) are easily prepared from amidines using the Graham oxidation with sodium hypohalites.^[^ ^29^ ^]^ The generality of the halogen exchange with other nucleophiles is well‐documented and enables access to a variety of substituents.^[^ ^30^ ^]^ Finally, an operationally facile, high‐yielding and broadly applicable one‐pot method (**route C**) converts unprotected amino acids into terminal 3*H*‐diazirines with hypervalent iodine in commercially available methanolic ammonia.^[^ ^31^ ^]^ Phenyliodine(III) diacetate (PIDA) is involved in three consecutive steps: it first promotes decarboxylation to generate an imine, which reacts with in‐situ‐generated iodonitrene (from 2 eq. PIDA) to form an iodinated diaziridine, precursor to the terminal 3*H*‐diazirine. An extension of this work includes an applicability to aromatic aldehydes and imines, as well as the use of cheaper *tert*‐butyl hypochlorite (*t*BuOCl) to prepare dialkyl diazirines in one pot from the corresponding ketones.^[^ ^32^ ^]^ The preparation of diazirines flanked by carbonyls and/or a halide is also described.^[^ ^33^, ^34^ ^]^

## 3 The Privileged Structure of Trifluoromethyl Aryl Diazirines (TADs)

**Figure 3:**
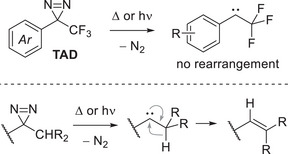
Top: Structure of 3‐trifluoromethyl‐3‐aryldiazirine (TAD) and the corresponding carbene. Bottom: General mechanism for the rearrangements of alkyl carbenes.

Among all the reported diazirine substitution patterns, the privileged structure of the 3‐trifluoromethyl‐3‐aryldiazirine (TAD), first described in 1980 by Brunner et al.^[^ ^6^ ^]^ (Figure 3, top), received dramatically more attention. Its appeal arises from its favorable photochemical properties, stability, ease of synthesis, and also because the trifluoromethyl group is not prone to the hydride and alkyl rearrangements that “plague the excited states of alkyl diazirines and carbenes” (Figure 3, bottom).^[^ ^24^, ^35^ ^]^ Indeed, the C─F bond (485 kJ/mol) is much stronger than a C─H (411 kJ/mol) or C─C bond (346 kJ/mol), thus preventing unwanted rearrangements in the popular TAD motif. Additionally, it has a reduced tendency for photoisomerization to its diazo isomer.^[^ ^6^ ^]^

**Figure 4:**
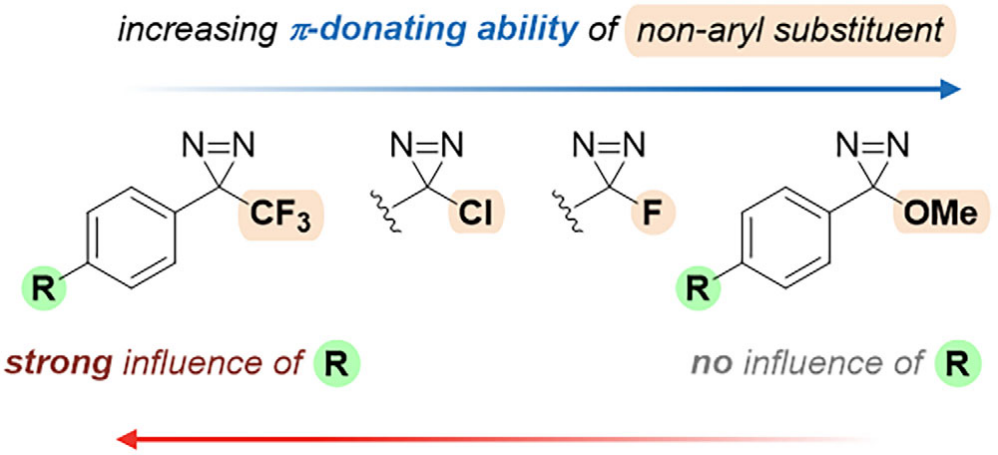
The second substituent on mono‐aryl diazirines modulates the influence of the electron density of the aromatic ring.

Beyond these advantages over alkyl diazirines, TADs also hold a privileged position with respect to other mono‐aryl diazirines. A 2021 study by Wulff et al.^[^ ^36^ ^]^ using differential scanning calorimetry (DSC) and computational methods has shed light on the much greater tunability of properties brought by the trifluoromethyl substituent (Figure 4). As described in later sections, this is rationalized by the capacity of the second, non‐aryl substituent to stabilize the evolving empty p‐orbital in the transition state via π‐donation. In other terms, the more π‐donating the second substituent is, the less contribution from the aromatic ring is allowed.

**Figure 5:**
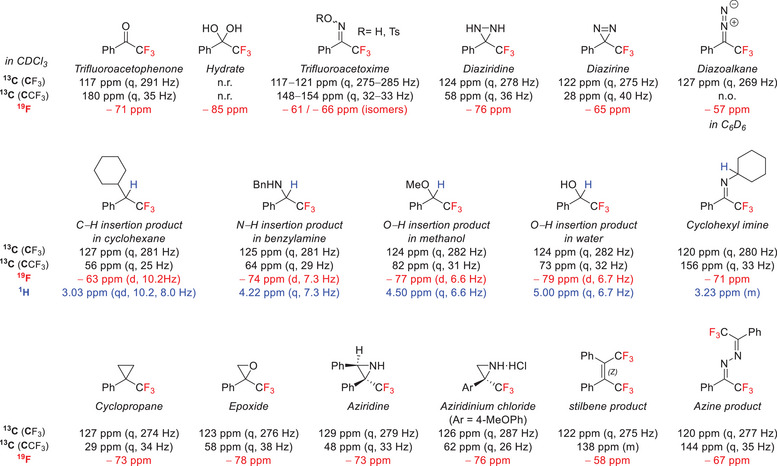
Chemical shifts in ^1^H, ^13^C, and ^19^F NMR for phenyl trifluoromethyl derivatives. n.r. = not reported; n.o. = not observed. (Tabulated values and references can be found in the Supporting Information.)

Finally, an extremely useful feature of TADs in a synthetic context is the advantageous NMR probe provided by the trifluoromethyl group. ^19^F NMR is a very sensitive analysis technique, both in intensity and resolution, and enables an easy diagnosis of the state of the diazirine moiety, its conversion to products or intermediates (see Figure 5).^[^ ^37^ ^]^ The chemical shift and coupling constant of adjacent carbons also help in the interpretation of ^13^C NMR spectra. Overall, these cumulated perks explain the historical success of Brunner's TAD structure in both the PAL and not‐PAL literature.

## 4 Carbene Generation

### 4.1 Direct Photolysis (Near‐UV)

**Figure 6:**
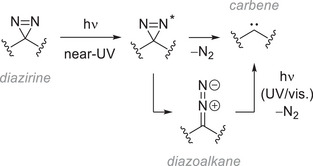
Photochemical activation of diazirines, including isomerization to the corresponding diazoalkane.

The direct photolytic activation of diazirines is the basis of PAL. As such, we send the reader to the many reviews focusing on this utilization of diazirines for extensive details of their properties.^[^ ^7^, ^8^, ^9^, ^11^ ^]^ Briefly, samples are irradiated by near‐UV light, which falls in the absorption band of the diazirine (Figure 6). The energized moiety can then either lose nitrogen gas and decompose to the corresponding carbene or rearrange into the diazo isomer.^[^ ^38^, ^39^, ^40^, ^41^ ^]^ The latter is also photolytically active, with an absorbance band in the near‐UV/visible region whose λ_abs_ and intensity strongly depend on the donor/acceptor character of the substituents.^[^ ^42^ ^]^ Thus, in general, continued irradiation ultimately leads to the same carbene upon loss of nitrogen.

**Figure 7:**
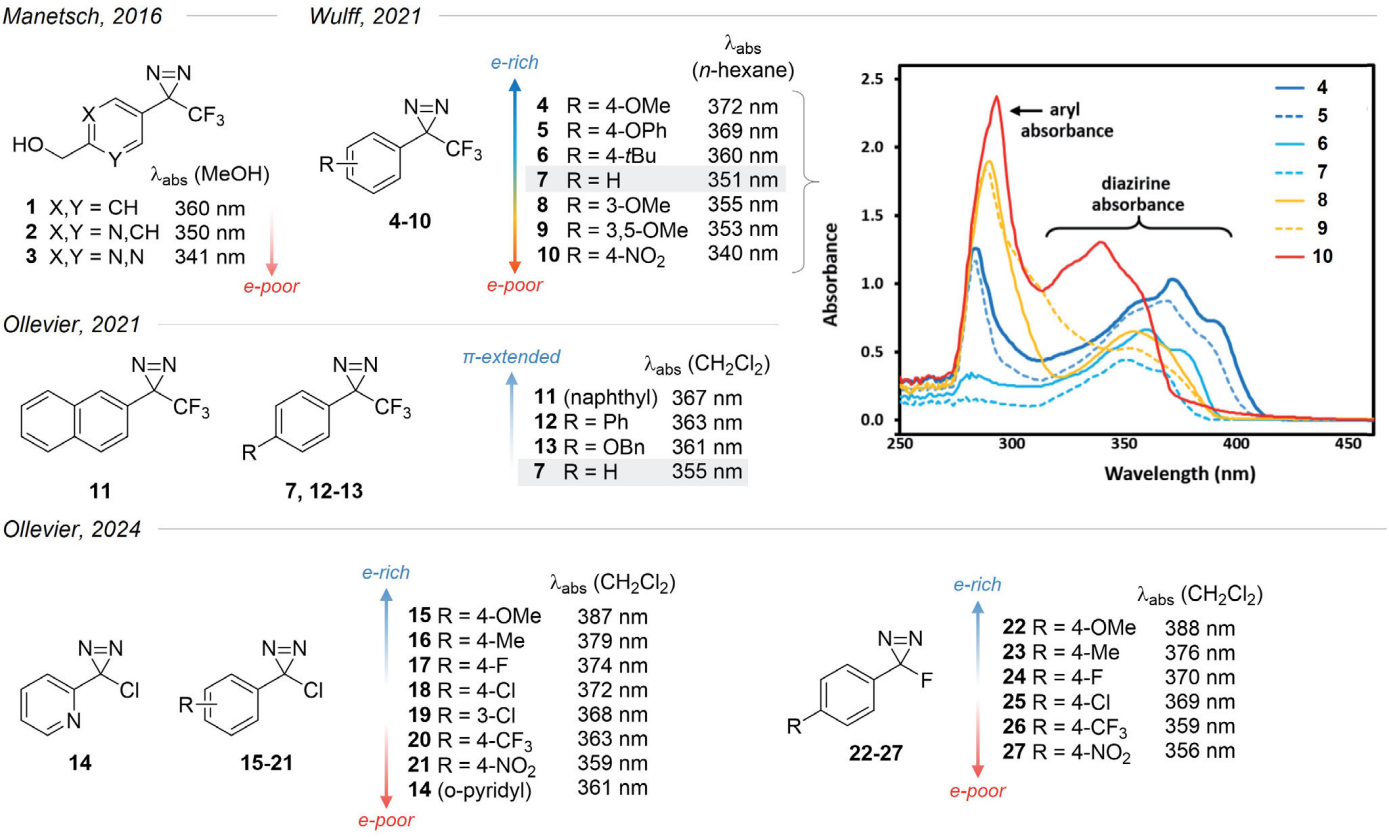
Variation in absorption wavelength for (hetero)aromatic diazirines.^[^ ^36^, ^43^, ^44^, ^45^, ^46^ ^]^ λ_abs_ is the apex of the absorbance band of the diazirine function and does not necessarily correspond to the maximum absorbance for the compound. The top right‐hand‐side panel was adapted from Wulff et al.^[^ ^36^ ^]^ (open‐access article under CC‐BY license).

Two studies from the Manetsch^[^ ^43^ ^]^ and Wulff^[^ ^36^ ^]^ groups focusing on TADs found that one can tune the absorbance of the diazirine moiety simply by adjusting the electron density on the neighboring aryl group: the more electron‐donating, the more red‐shifted (Figure 7). By replacing the phenyl ring with an electron‐poor pyridine or pyrimidine, Manetsch et al. decreased the local λ_abs_ (corresponding to the diazirine absorbance) by ca. 10 nm and 20 nm, respectively.^[^ ^43^ ^]^ The Wulff group could achieve a similar result by adding a strongly electron‐withdrawing *para*‐nitro group on the classical Brunner's TAD, whereas a *para*‐methoxy group pushed the λ_abs_ up to 372 nm.^[^ ^36^ ^]^ The latter compound and the *para*‐phenoxy derivative had a reasonable absorbance even above 400 nm, enabling their activation by visible light. Substitution on the meta position had little to no impact. Of note, Ollevier et al.^[^ ^44^ ^]^ managed to activate diazirines for cyclopropenation with alkynes (see below) using purple LEDs at 420 nm in a flow setup, although this wavelength range only applied to a subset of diazirines with extended π‐conjugation. The same group later investigated halodiazirines, and their measurement of UV/Vis spectra shows similar trends spanning 30 nm, with a small bathochromic shift due to a better π‐donation from the halogen (F, Cl).^[^ ^45^, ^46^ ^]^

**Figure 8:**
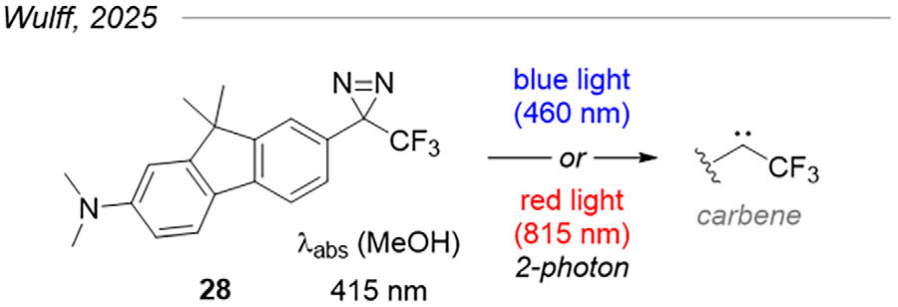
Model fluorene‐diazirine conjugate.^[^ ^47^ ^]^

Taken together, these results suggest that the aromatic ring in TADs is somehow electronically conjugated to the diazirine moiety, a counterintuitive fact considering its apparent tetrahedral geometry. This triggered the interest of the Wulff group: they assembled key pieces of evidence (structural, photophysical, and computational) in a recent article supporting that a diazirine's central carbon is actually sp^2^‐hybridized.^[^ ^47^ ^]^ Their conclusions led them to design the model fluorene‐diazirine conjugate **28** (Figure 8) that is activated by direct blue light (> 450 nm) or by two‐photon absorption with red light (860 nm), two landmark innovations in the diazirine field.

**Figure 9:**
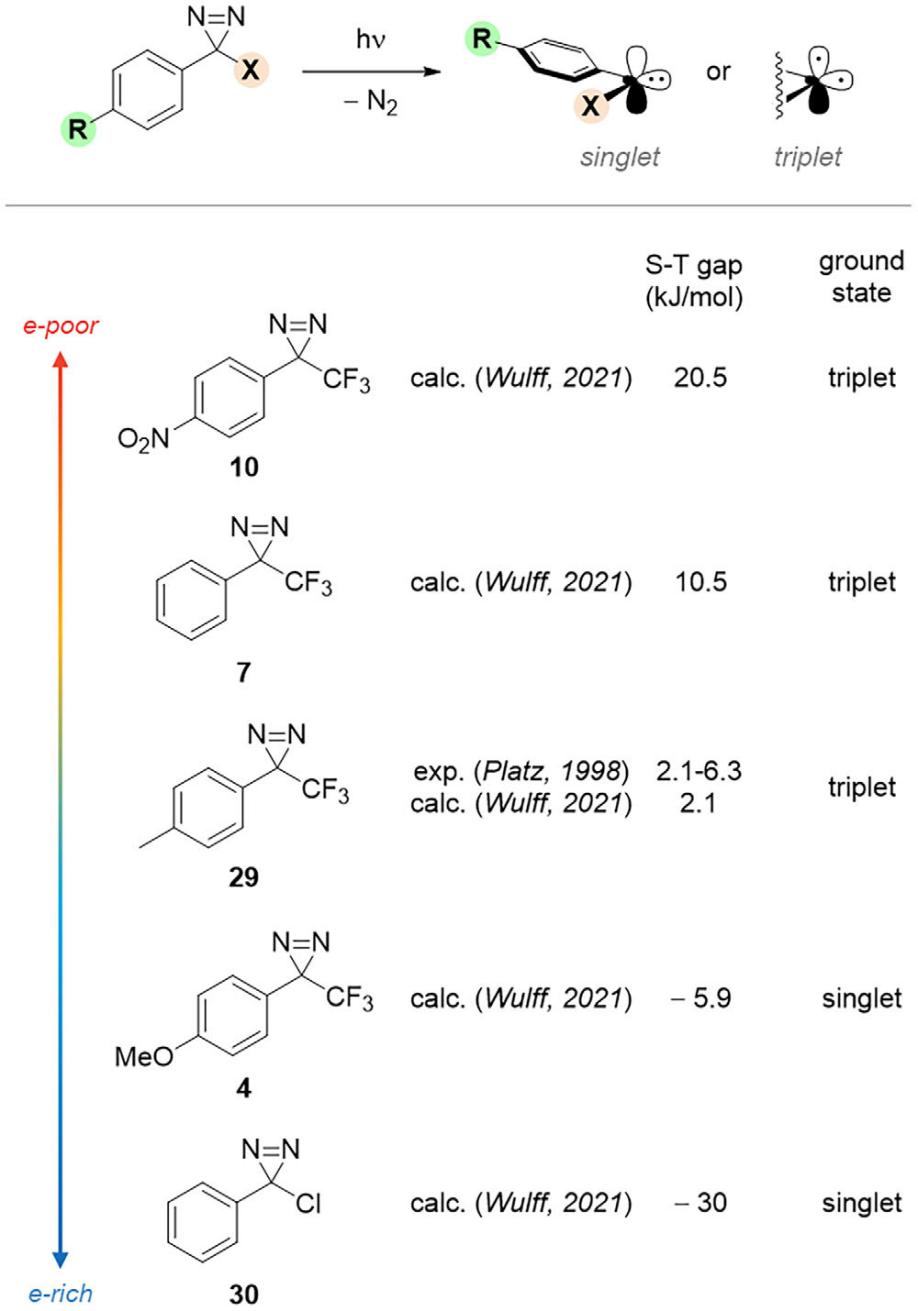
Substituents strongly influence the electronic state of diazirine‐born carbenes.

The electronic state of photochemically generated, diazirine‐born carbenes (i.e., singlet or triplet) has important consequences in terms of downstream reactivity, as well as the singlet‐triplet (S‐T) gap. Simplistically, a singlet carbene will engage in two‐electron processes whereas a triplet carbene will behave as a diradical. Laser flash photolysis (LFP) studies by Platz et al. showed that the *p*‐tolyl(trifluoromethyl) carbene generated by near‐UV irradiation of diazirine **29** (Figure 9) has a triplet ground state, but with an “extremely small” S‐T gap of 0.5‐1.5 kcal/mol (2.1‐6.3 kJ/mol) actually allowing reactivity primarily via its low‐lying singlet state.^[^ ^35^ ^]^ Density functional theory (DFT) calculations by Wulff, DiLabio et al. are in agreement with this experimental result (2.1 kJ/mol) and highlight the strong influence of *para* substituents of TADs on the S‐T gap of the corresponding carbene (Figure 9).^[^ ^36^ ^]^ Particularly, Brunner's TPD **7** appears to have a significantly higher S‐T gap (10.5 kJ/mol). On the contrary, carbenes obtained from monoaryl diazirines bearing a strong π‐donating second, non‐aryl substituent (X = Cl, F, OMe) are calculated to have a singlet state > 20 kJ/mol more stable than the triplet, independent of aryl electronics.

### 4.2 Thermolysis

**Figure 10:**
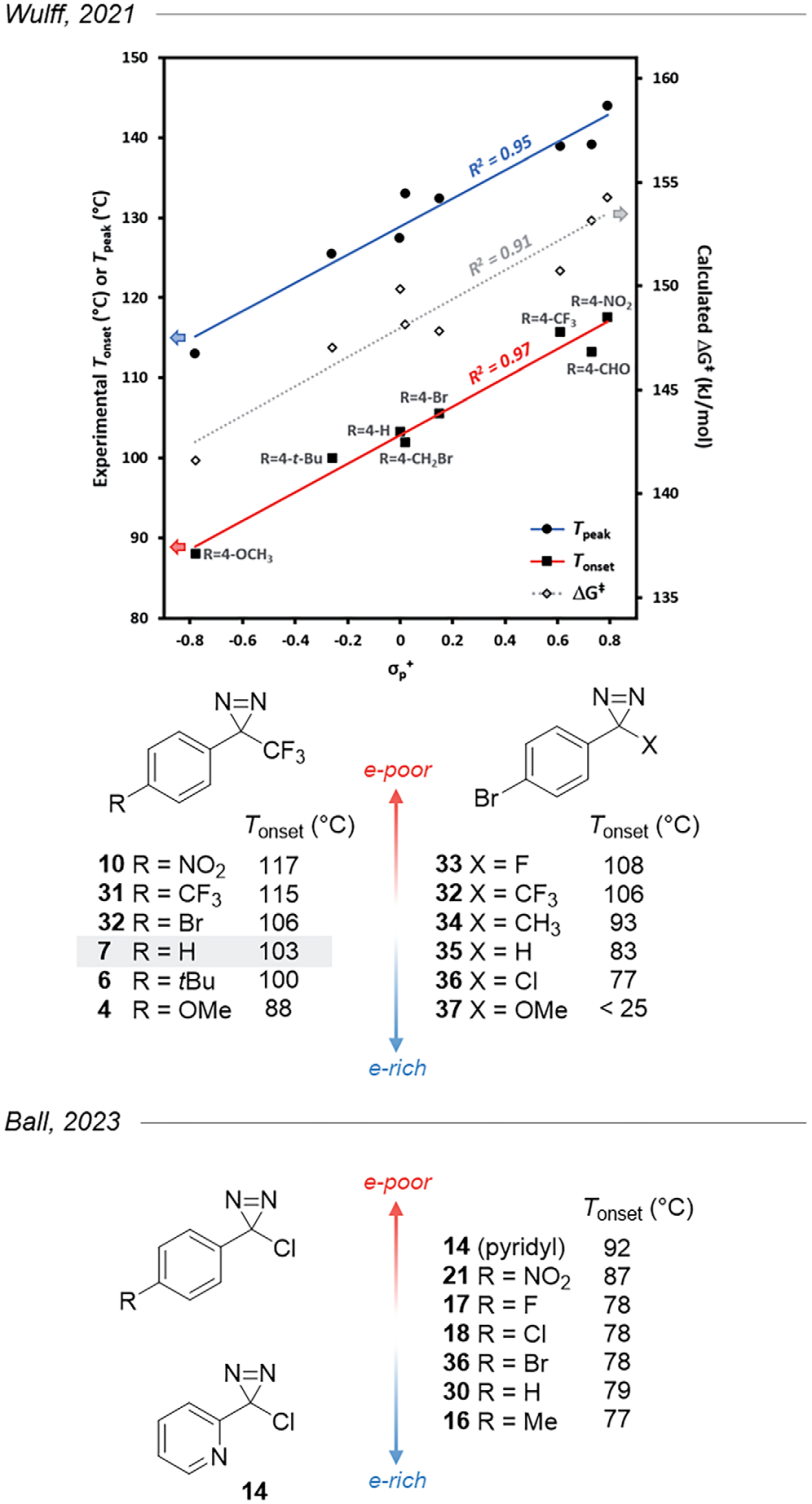
Top: Variation in TAD activation temperatures and energies with the Hammett parameter σ_p_^+^.^[^ ^48^ ^]^ The strong correlations and positive slopes are indicative of carbocation character in the transition state resulting from diazirine activation. Reproduced from Wulff et al.^[^ ^36^ ^]^ (open‐access article under CC‐BY license). Bottom: Selected examples of TADs^[^ ^36^ ^]^ and aryl diazirines,^[^ ^50^ ^]^ illustrating that electron‐donating aryl substituents lower their activation temperatures.

Most diazirines are conveniently stable at room temperature but can be activated thermally. Until recently, little systematic work had reported onset temperatures and reaction yields after heating diazirines.^[^ ^12^ ^]^ Based on DSC studies, Wulff et al.^[^ ^36^ ^]^ identified a strong correlation between the electron density of TADs and their decomposition temperatures. In particular, the onset and peak temperatures (determined from the exotherm recorded by DSC) correlated very well with Brown's σ_p_^+^ parameters^[^ ^48^ ^]^ of substituents located across the aromatic ring from the diazirine moiety, as did the calculated free energy of activation toward carbene formation (Figure 10). Strikingly, the onset temperature for TADs could be easily tuned over a 30 K range, from 88 °C for 4‐OMe to 118 °C with 4‐NO_2_. In the same work, a similar analysis shows that replacing the CF_3_ group with less electron‐withdrawing substituents (e.g., Cl; see compounds **32** and **36** in Figure 10) can lower the activation temperature by the same 30 K magnitude. At the extreme, the room‐temperature instability of methoxy aryl diazirines (such as **37**) was observed in 1987 by diazirine and carbene specialist R. Moss and his group on methoxy phenyl diazirine.^[^ ^49^ ^]^ More recently, Ball and coworkers have performed another DSC‐based study on aryl chlorodiazirines that showed a similar trend, yet with a narrower temperature range (Figure 10, bottom).^[^ ^50^ ^]^

Altogether, the understanding of structure‐reactivity of diazirines brought by these systematic studies is likely to have a major impact on uses in materials science, where activation temperature needs to be adjusted to the substrate and/or the application. As of today, and to the best of our knowledge, there seems to be no calorimetry data allowing a similar analysis on alkyl diazirines. The intermediacy of isomeric diazoalkanes during thermolysis of diazirines, which was clearly demonstrated by Jennings and Liu in a 1976 report on butyl phenyl diazirine,^[^ ^51^ ^]^ remains a complex question for which we direct the reader to a 2011 comprehensive review on diazo/diazirine isomerism by S. Korneev.^[^ ^39^ ^]^

### 4.3 Electrolysis

**Figure 11:**
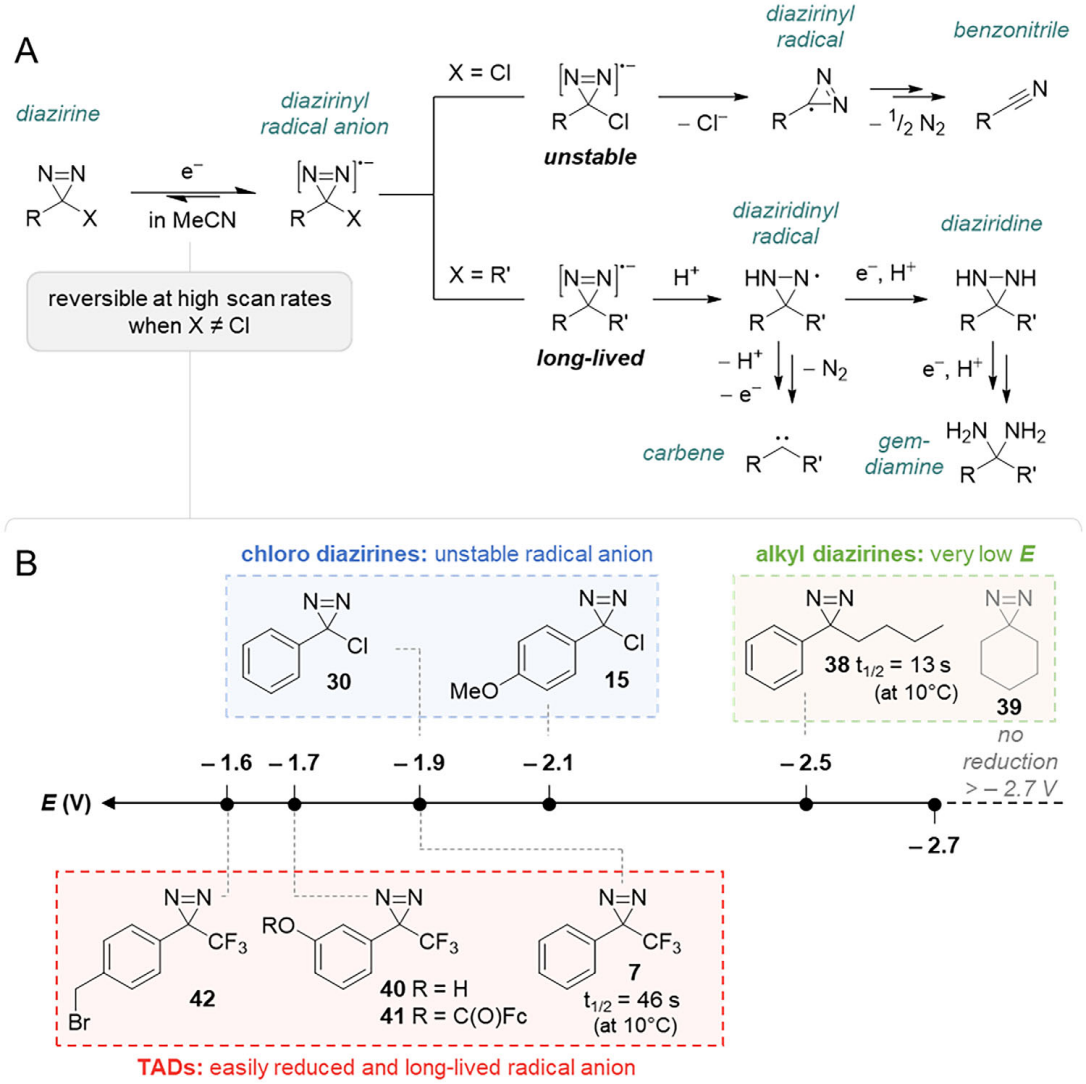
A) Scheme representing the electrochemical reduction of various diazirines and their downstream pathways. B) Reduction potentials of studied diazirines, indicated versus Ag/AgCl. The indicated half‐lives were measured in MeCN + 0.1 M Et_4_N·ClO_4_ at 10 °C in the absence of a proton donor. For clarity, the values represented on the potential axis are approximations, given the variety of reference electrodes and conditions used in the studies. An inventory of the exact, reported values and the experimental parameters can be found in the Supporting Information.

Diazirines are redox‐active species that can be triggered electrochemically. Starting in 1982, Liu et al. were the first to explore the electrochemical behavior of aryl diazirines.^[^ ^52^ ^]^ Using cyclic voltammetry, they observed one‐electron reduction of aryl chlorodiazirines (**30**, **15**) and butyl phenyl diazirine **38** with more than 0.5 V of difference in potential (Figure 11). Furthermore, they could not observe the reduction of pentamethylene diazirine **39**, even at −2.7 V in MeCN + 0.1 M NEt_4_ClO_4_ (vs. Ag/AgNO_3_ at a vitreous carbon electrode). These observations indicate that electron‐rich and —more particularly— alkyl diazirines are harder to reduce (Figure 11B). Whereas this reduction is irreversible for chlorodiazirines and leads to benzonitrile after loss of chloride, the radical anion of butyl phenyl diazirine **38** could be produced reversibly in aprotic medium. In the presence of minute amounts of a proton donor, this radical anion is reduced further down to the corresponding diaziridine, whereas reduction in acidic protic media yields the corresponding ketone via full reduction to the *gem*‐diamine and hydrolysis (Figure 11A).^[^ ^53^ ^]^ In a follow‐up study,^[^ ^54^ ^]^ Liu et al. characterized the diazirinyl radical anions of butyl phenyl diazirine **38** and Brunner's TPD^[^ ^6^ ^]^ **7** and measured their respective half‐lives (t_1/2_ = 13 s and 46 s at 10 °C, resp.) (Figure 11B). The trifluoromethyl substituent greatly facilitates the reduction and participates in the stabilization of the radical. Nonetheless, their analysis of the EPR spectra led them to propose that the additional electron is localized mainly on the two nitrogen atoms for both compounds. Of note, unrelated work on TADs **40,41** was consistent with the measured reduction potentials.^[^ ^55^ ^]^

It was found later that ketone‐photosensitized oxidation of diaziridines led to the generation of carbenes, presumably via their diaziridinyl radical, the alleged intermediate in diazirine electrochemical reduction.^[^ ^56^ ^]^ Taken together, these reports inspired Steele et al. in their design of multivalent PAMAM‐based diazirine electro‐curable adhesives.^[^ ^14^ ^]^ They found that a potential below − 1.6 V (vs. Ag/AgCl) triggered the activation of a typical TAD motif (*para*‐bromomethyl‐TPD **42**) grafted on the poly(amidoamine) core in a phosphate‐buffered saline (PBS), in turn promoting crosslinking in the adhesive layer through carbene insertion. The exact mechanism for carbene generation and the subsequent X─H insertion (X = C, O, N, etc.) is still unclear, although the intermediacy of diazoalkanes and the crosslinking through azine motifs were ruled out in a later study by the same group using alternative current (AC) rather than direct current (DC).^[^ ^57^ ^]^ Nonetheless, the authors acknowledge that other crosslinking mechanisms cannot be excluded.

### 4.4 Photocatalysis (Blue Light)

**Figure 12:**
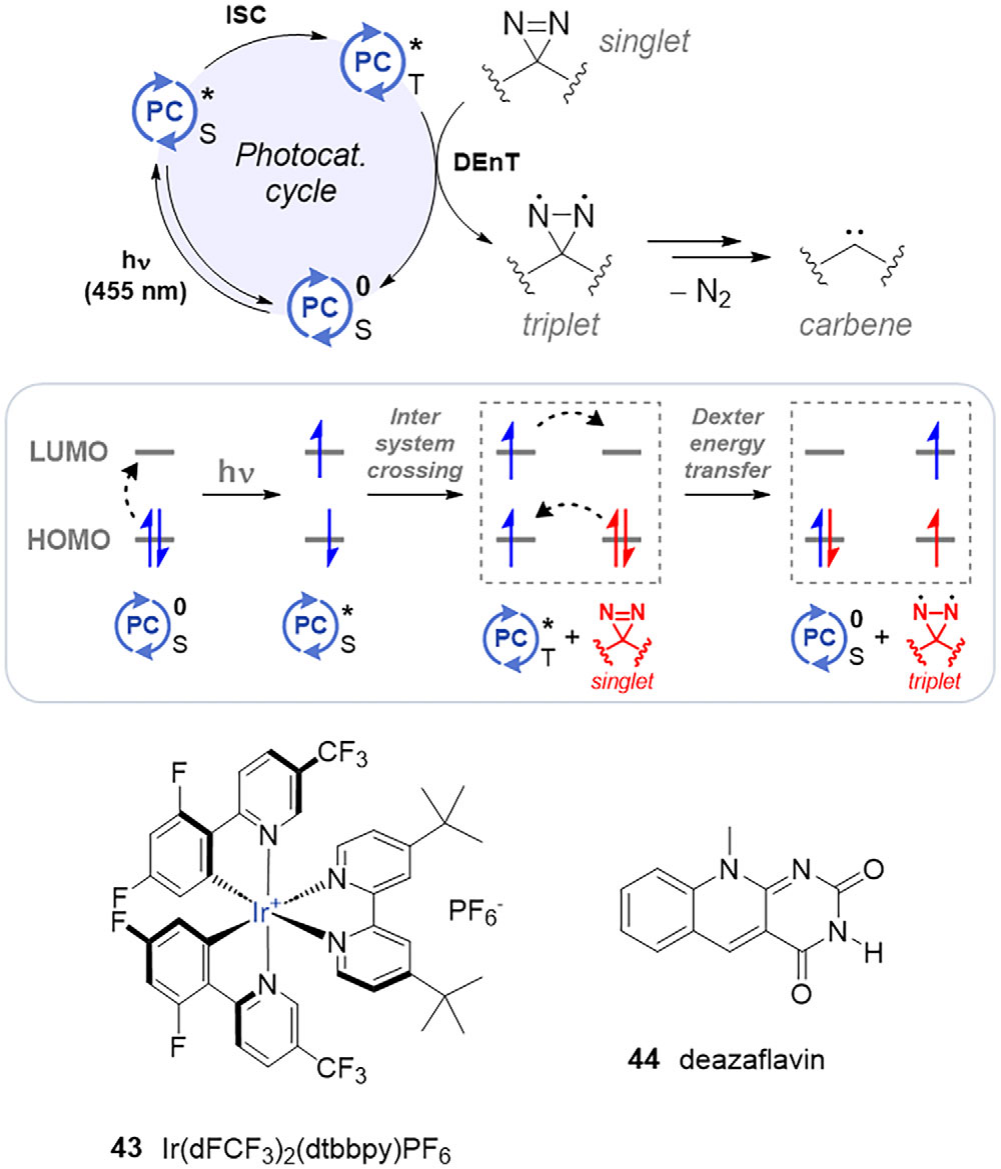
Top: Overview of photocatalytic activation of diazirines with blue light. Middle: Schematic representation of electron population in frontier orbitals of photocatalyst and diazirine during activation by DEnT. Bottom: iridium‐based and deazaflavin photocatalysts.

In a study from 2020, MacMillan et al. uncovered a new method to activate diazirines with blue light and a photosensitizer.^[^ ^58^ ^]^ Upon irradiation, the iridium photosensitizer **43** is energized in its triplet excited state (≥ 60 kJ/mol above its ground state), at which point it triggers diazirine decomposition via a Dexter energy transfer (DEnT) (Figure 12). This latter process only occurs at very short distances (typically < 1 nm), thereby spatiotemporally localizing carbene generation in the near vicinity of the photosensitizer. In the study, the nascent carbene could then react with neighboring X─H bonds, for instance, to attach diazirine‐armed biotin onto target nearby biomolecules for later detection with streptavidin‐based Western blot. By conjugating the photosensitizer to specific antibodies, they harnessed the spatiotemporal resolution of the carbene generation to map the protein microenvironment of immune cells.

Prior to their biology assays, they demonstrated the feasibility of their approach on a small set of substrates, consisting of TADs bearing various electron‐withdrawing and ‐donating groups, with very high yields (75–100%) of O─H insertion in aqueous medium. Such mild reaction conditions, allied to the potential of spatiotemporal control over carbene generation, are bound to generate a lot of interest from various fields of science. Indeed, Steele et al. successfully integrated this activation mode into their diazirine‐armed dendrimer adhesives,^[^ ^59^ ^]^ thereby demonstrating the applicability of this method to materials science.

Shortly before we wrapped up this review, Hackenberger et al. released a preprint suggesting that deazaflavin **44** (Figure 12, bottom), an organic cofactor with a triplet energy of 59.6 kcal/mol, can also trigger TAD decomposition into carbenes under blue‐light irradiation.^[^ ^60^ ^]^

### 4.5 (Ineffectiveness of) Metal Catalysis

Unlike diazo compounds, diazirines are generally stable toward transition metals. To the best of our knowledge, there is no clear report of generic formation of metal carbenes from diazirines without any other activation mode favoring the extrusion of N_2_ (incl. thermolysis of electron‐rich diazirines). A series of experiments by Chaloner et al. in the 1980s were conflicting: whereas an early report^[^ ^61^ ^]^ first suggested insertion of W(CO)_5_ or Cr(CO)_5_ into a C─N bond of methoxy phenyl diazirine (notably electron‐rich), a later one demonstrated that carbene generation by thermolysis (albeit at low temperature) was a key step.^[^ ^62^ ^]^ In 2000, Moss also noted a catalytic effect of AlCl_3_ on the decomposition of diazirines via an interaction with the nitrogens’ lone pairs.^[^ ^63^ ^]^ Some recent reports discussed later in this review hint at a combination of metal activation and steric pressure for isomerization of diazirines to diazoalkanes.

## 5 Diazirines as Carbene Precursors

The main drive for the development of diazirine chemistry has certainly been the ease of generating free carbenes bearing a wide variety of substituents upon simple photolysis of (generally) stable reagents. As we are modestly attempting to give here an overview of diazirines’ potential in synthesis, we are well aware of the quality and amount of work published by pioneers in carbene studies such as R. Moss, M.T.H. Liu, and M.S. Platz, among others. Their findings are related in several accounts^[^ ^30^, ^64^, ^65^, ^66^, ^67^, ^68^ ^]^ spanning four decades, to which we direct the reader as they exceed the scope of this review. In the present section, we will collate approaches that use the carbenic reactivity of diazirines for synthetic purposes.

### 5.1 Glycosylidene Diazirines

One of the most cohesive and substantial studies of diazirine outside of PAL is the work of Vasella et al. on the use of glycosylidene carbenes in carbohydrate chemistry. Following the initial conceptualization in 1989,^[^ ^69^ ^]^ they regularly published their systematic progress over almost 15 years in a series of more than 30 research articles in *Helv. Chim. Acta* (and a few elsewhere). The key lessons of this major contribution were summarized in a book chapter^[^ ^70^ ^]^ of which we will only give a brief summary here.

**Figure 13:**
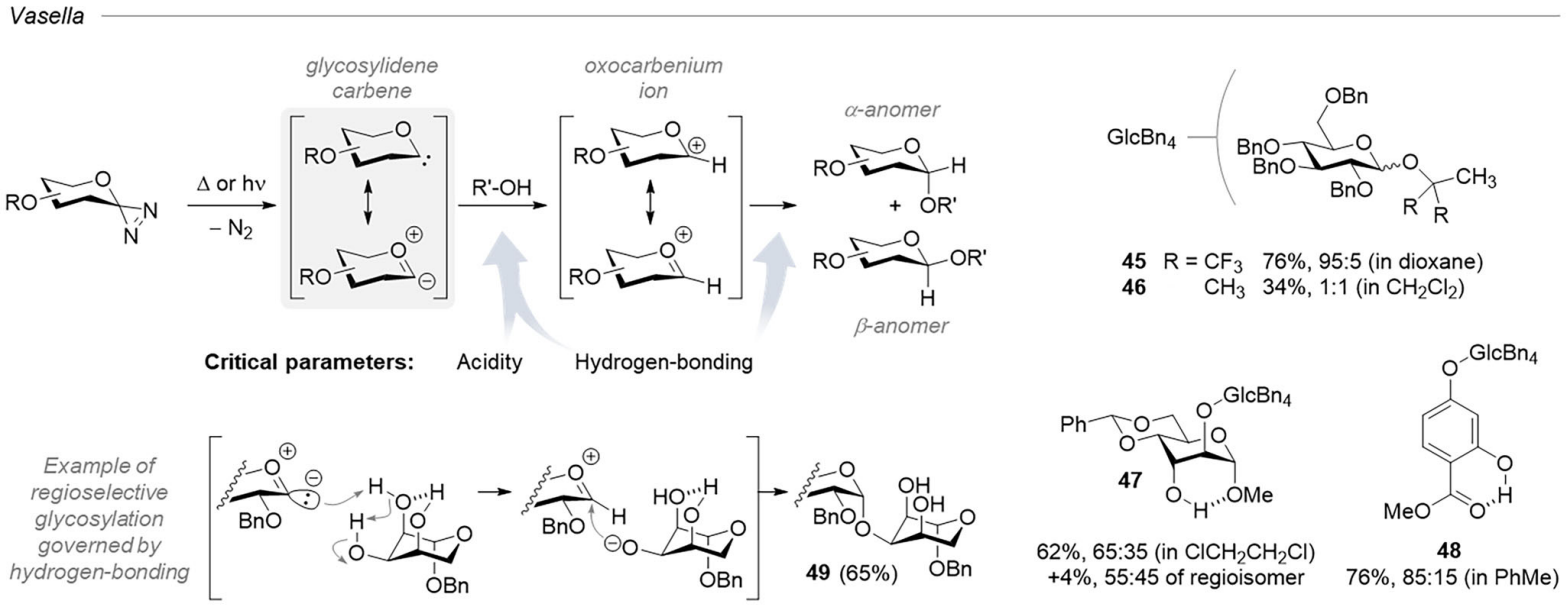
Concept of glycosylation with glycosylidene carbenes and alcohols, and key examples illustrating the influence of acidity and hydrogen bonding over regioselectivity.

The concept stemmed from the recognition that a carbene located at the anomeric center of a glycosyl residue could insert into the O─H bond of an alcohol (potentially another carbohydrate residue), leading to a new glycosidic linkage (Figure 13). Though less reactive than archetypical methyl methoxy carbene,^[^ ^71^ ^]^ glycosylidene carbenes do react with alcohols via a mechanism involving their protonation to afford a canonical oxocarbenium onto which an alkoxide can add. Based on their extensive studies, Vasella and coworkers exposed key parameters governing this reactivity. Primarily, the reaction rate is strongly correlated with acidity, meaning fluorinated alcohols and phenols are prime substrates for this approach because they are deprotonated faster. Consequently, hydrogen bonding plays a critical role, as it modulates the acidity of hydroxy groups. Solvation of the ion pairs is also an important parameter, contrary to sterics, which seem to have little impact.

The synthetic utility of this fascinating reactivity is to be moderated in regard to the reported challenging access to the glycosylidene diazirines (short lifetime at room temperature) and the modest anomeric selectivity afforded in most cases. Nonetheless, “*regioselective glycosidation of diols and triols by diazirines is feasible and often strikingly successful. Its course can be predicted, even if the factors are complex, if the hydrogen bonds in the glycosyl acceptor are known*.”^[^ ^70^ ^]^ Conversely, glycosylidene carbenes can be used as reactive probes to investigate hydrogen‐bonding networks in polyols.

Glycosylidene carbenes also react with a variety of reagents via cyclopropanation, epoxidation, and insertions, and again we redirect the reader to Vasella's work. Of note, a 2024 report by Gang He et al. described the in situ generation of glycosylidene carbenes via copper‐catalyzed oxidation of the corresponding diaziridines and their reaction with boronic esters to access boro‐ketosides that could be further derivatized into a series of *C*‐glycosides.^[^ ^72^ ^]^

### 5.2 Diazo Precursor and Cyclopropanation

**Figure 14:**
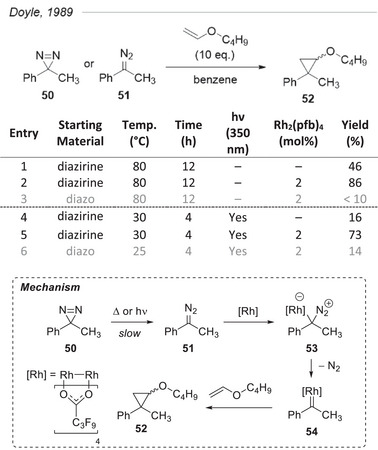
Usage of diazirine **50** as a precursor of diazo for its cyclopropanation with butyl vinyl ether. The higher yields obtained in the presence of 2 mol% Rh_2_(pfb)_4_ support a mechanism where the diazoalkane **51** generated from the slow decomposition of diazirine **50** is trapped as a rhodium(II)‐carbene.

A leading figure in the field of carbene chemistry,^[^ ^73^ ^]^ Doyle recognized early on the potential of diazirines as a more stable precursor of carbenoids than diazoalkanes.^[^ ^74^ ^]^ In work from 1989, his group took advantage of the reported thermal^[^ ^51^ ^]^ and photochemical^[^ ^40^ ^]^ isomerization of 3‐alkyl‐3‐aryldiazirines to their corresponding diazoalkanes to circumvent typical side reactions of the latter: formation of azines or carbene dimers, rearrangement to alkenes by 1,2‐hydride shift, etc. The slow conversion of diazirine **50** to diazo **51**, either by heating (80 °C) or UV irradiation (350 nm), allowed the controlled formation of the desired rhodium(II)‐carbenoid **54**, which could in turn undergo cyclopropanation with butyl vinyl ether (Figure 14). Good yields of the desired cyclopropane support the proposed mechanism. The authors stress that the rate‐limiting production of the diazo compound, thus preventing the formation of by‐products, is key to the observed selectivity toward cyclopropanation.

### 5.3 Pd(0) Cross‐Couplings

**Figure 15:**
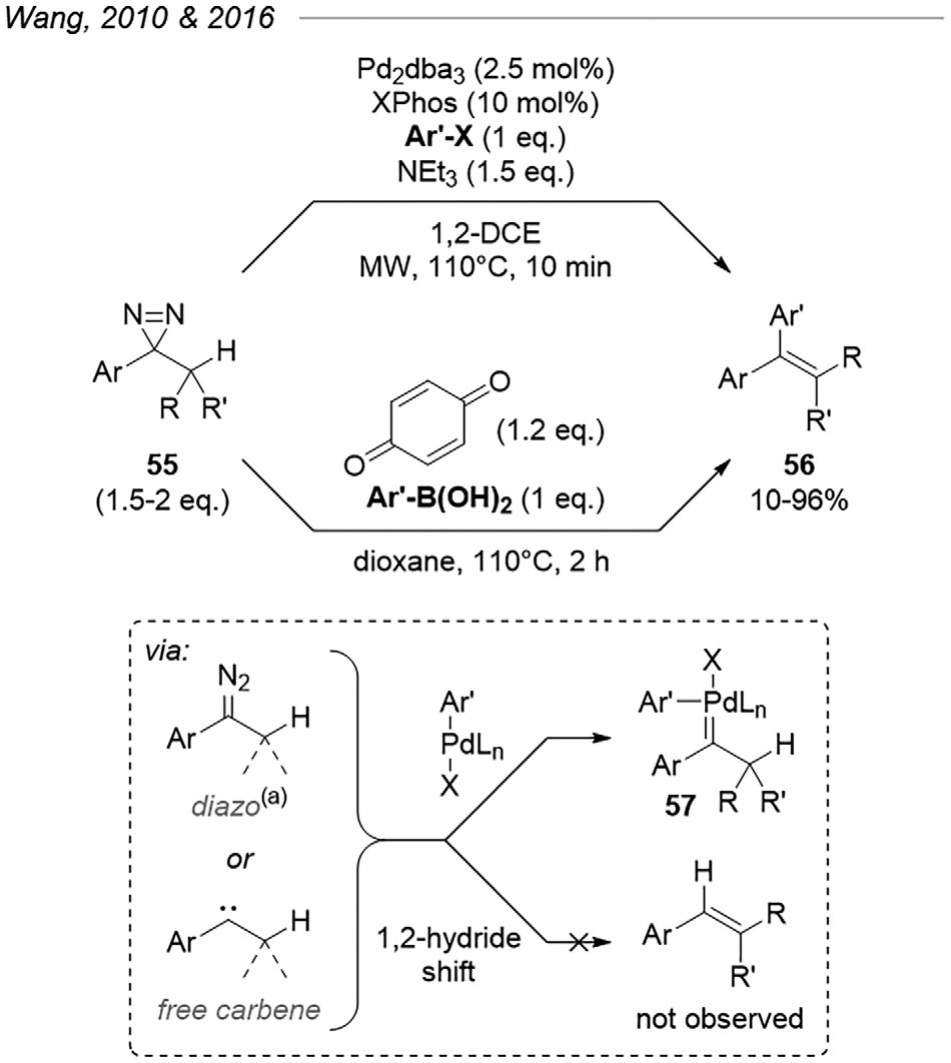
Top: Pd(0)‐catalyzed, microwave‐assisted cross‐couplings of aryl alkyl diazirines with aryl halides.^[^ ^75^ ^]^ Bottom: Metal‐free oxidative cross‐coupling of aryl alkyl diazirines with aryl boronic acids.^[^ ^76^ ^] (a)^ Diazo isomer was ruled out as an intermediate in the metal‐free cross‐coupling.

Despite this precedent from Doyle et al., twenty years passed before Wang et al. reported a microwave‐assisted, Pd(0)‐catalyzed cross‐coupling of 3‐alkyl‐3‐aryldiazirines **55** with aryl halides (Figure 15).^[^ ^75^ ^]^ Thermal activation of diazirines at an elevated temperature (110 °C) generated either free carbenes or isomeric diazo compounds that reacted with the Pd(II) species resulting from oxidative addition to form **57**, which underwent successive carbene migratory insertion and β‐hydride elimination, yielding the desired alkene **56** and regenerating the catalyst after neutralization of hydrohalic acid. They later found that such couplings can be performed without participation of a metal when using arylboronic acids and benzoquinone in place of aryl halides.^[^ ^76^ ^]^

On this occasion, they also could exclude the intermediacy of diazoalkanes in the mechanism, based on a control reaction with a metalated tosylhydrazone (precursor to diazo via thermal activation) instead of a diazirine. This fact conflicts with earlier observations by Doyle and others (see above) and may suggest the involvement of free carbenes, potentially via alternative pathways catalyzed by Lewis acids (Pd complex or boronic acids). Nonetheless, the fact that diazirines activated at 110 °C still reacted cleanly with either the palladium catalyst (only 5 mol% Pd)^[^ ^75^ ^]^ or the boronic acids^[^ ^76^ ^]^ —while avoiding the nonproductive 1,2‐hydride shift pathway— speaks against the generally assumed intractability of this class of species.

### 5.4 Enzymatic Cyclopropanation of Acrylates

**Figure 16:**
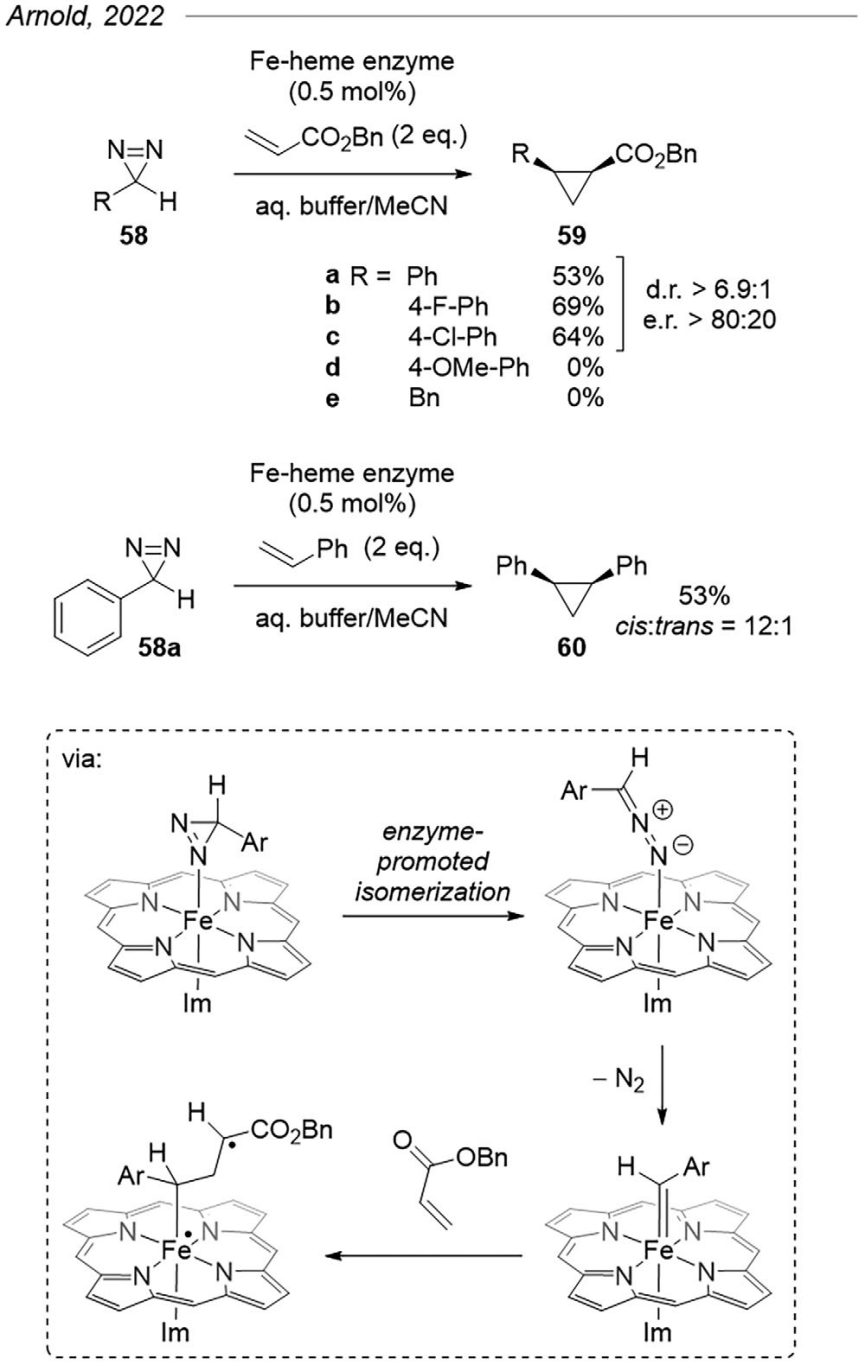
Enzymatic cyclopropanation of benzyl acrylate and styrene by Arnold et al.^[^ ^77^, ^78^ ^]^

In a study from 2022, Arnold et al. demonstrated the activation and control of a simple aryl diazirine with an Fe‐heme enzyme (*Aeropyrum pernix* protoglobin, *Ape*Pgb) obtained by directed evolution to achieve the stereoselective cyclopropanation of benzyl acrylate (Figure 16, top).^[^ ^77^ ^]^ Electron‐neutral 3*H*‐3‐phenyl diazirine **58a** served as the optimization substrate, reaching 53% yield and modest enantio‐ and diastereomeric ratios. The optimized enzyme *Ape*Pgb GLAVRSQLL also accommodated electron‐poor aryl diazirine substrates in slightly improved yields and stereoselectivities, without requiring any optimization. Meanwhile, it proved ineffective with electron‐rich aryl *p*‐methoxyphenyl diazirine or benzyl diazirine. Cyclopropanation of styrene was also achieved with phenyl diazirine in 61% yield, but attempts at C─H, N─H, and Si─H insertions provided low or no yields (not shown here).

A follow‐up computational study^[^ ^78^ ^]^ using a combination of DFT, quantum mechanics/molecular mechanics (QM/MM), and molecular dynamics (MD) approaches allowed them to support their initial hypothesis of a diazirine‐to‐diazo isomerization as the first step of the enzymatic transformation (Figure 16, bottom). This work also revealed that cyclopropanation takes place via a stepwise, diradical mechanism, and that the diastereoselectivity is under steric control.

### 5.5 Diazirine Activation with a Sterically Hindered Copper Complex

**Figure 17:**
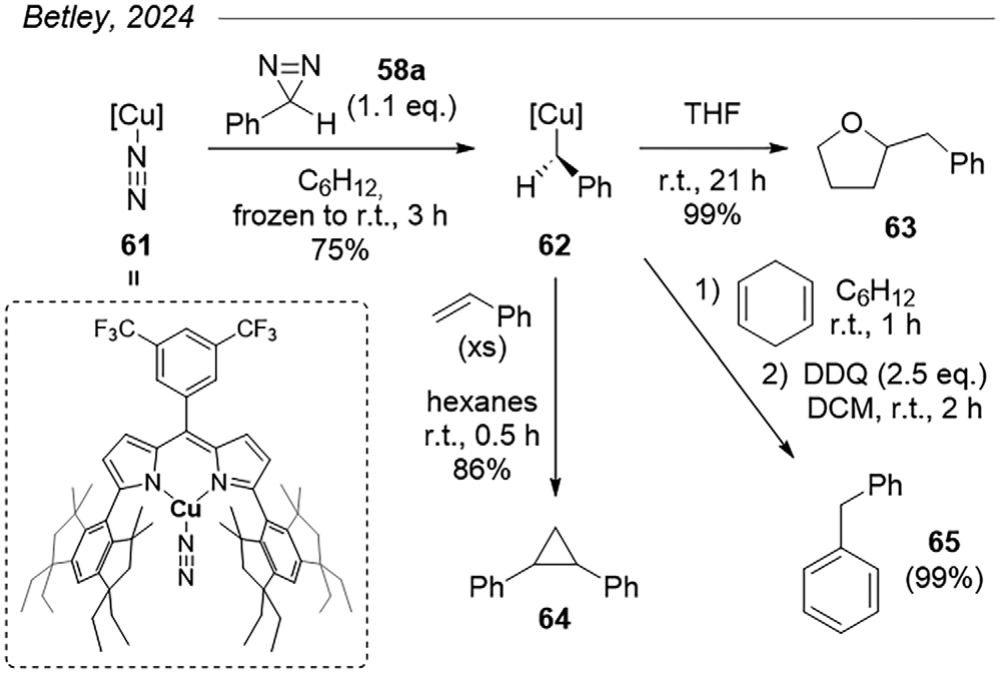
Recent report of the formation of a copper benzylidene complex from a diazirine below room temperature.

Very recently, Betley et al. reported the formation of an isolable copper benzylidene complex from 3*H*‐3‐phenyl diazirine **58a** below room temperature (Figure 17).^[^ ^79^ ^]^ This is a surprising result given the general stability of diazirines in the presence of transition metals. No explanation for this activation was mentioned, but interestingly, no reaction was observed when replacing the hexahydroindacene fragments by less sterically demanding triphenylaryl groups (not shown here). Whereas the authors invoke a deactivation due to competitive binding of flanking aryl groups to the Cu center, one can also wonder if the combined electrophilic activation by copper and steric pressure exerted by the complex **61** do not constitute a synergistic trigger for diazirine decomposition. This would then align with the enzyme‐promoted diazirine‐to‐diazo isomerization posited by Houk and Arnold and discussed just above. The formed complex **62** displays electrophilic C─H insertion and cyclopropanation reactivity that the authors ascribe to a combination of electronic and steric factors.

### 5.6 In Situ Cycloadditions

**Figure 18:**
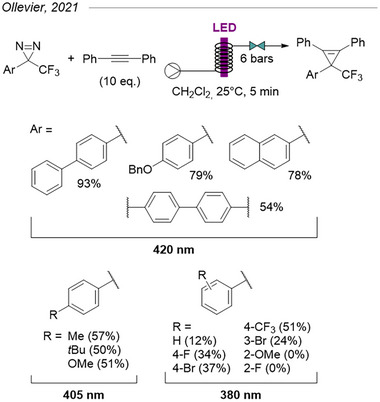
Cyclopropenation of diphenylacetylide with TADs under direct photolytic activation in flow.

The work described above mostly relies on control of diazirine‐born carbenes by transition metals, but several synthetic applications of diazirines do not require such control. In 2021, Ollevier et al. demonstrated the feasibility of uncatalyzed, metal‐free cyclopropenations of alkynes with TADs by direct photolytic activation.^[^ ^44^ ^]^ Free carbenes were generated using low‐energy near‐UV and purple LEDs (380–420 nm) in a flow reactor in the presence of an excess of alkyne (10 equiv.) with an optimal residence time of only 5 minutes (Figure 18). The scope seemed quite wide for both internal and terminal alkynes (not shown here), although yields for the latter class were diminished due to competitive C─H insertion. Despite its remarkable simplicity, one limitation of this method resides in the scope of usable diazirines: only TADs were described, and activation under visible light remains limited to a small subset of π‐extended aromatics due to red‐shifting of the diazirine absorbance (*vide supra*). Electron‐rich mono‐aryls would still provide acceptable yields under 405 nm, but other substitution patterns required UV irradiation and provided only moderate to low yields. The authors rationalized this by the side reactions promoted by the higher‐energy irradiation.

**Figure 19:**
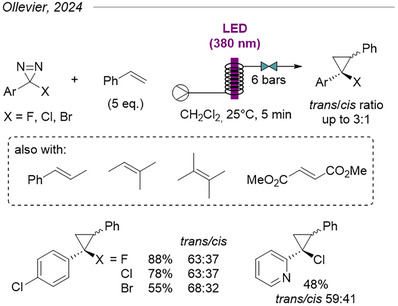
Cyclopropanation of alkenes with halodiazirines under direct photolytic activation in flow.

The same group later applied their flow system to the cyclopropanation of various alkenes with halodiazirines (Figure 19).^[^ ^45^, ^46^ ^]^ Although they report activation at 405 nm for *p*‐nitrophenyl halodiazirines, their conditions generally employ 380 nm irradiation across a range of aryl halodiazirines featuring either electron‐withdrawing or electron‐donating substituents. Modest to little diastereoselectivity was observed for most compounds, and diastereomers could not be separated.

### 5.7 P─H Insertion of Diazirine‐Born α‐Halocarbenes

**Figure 20:**
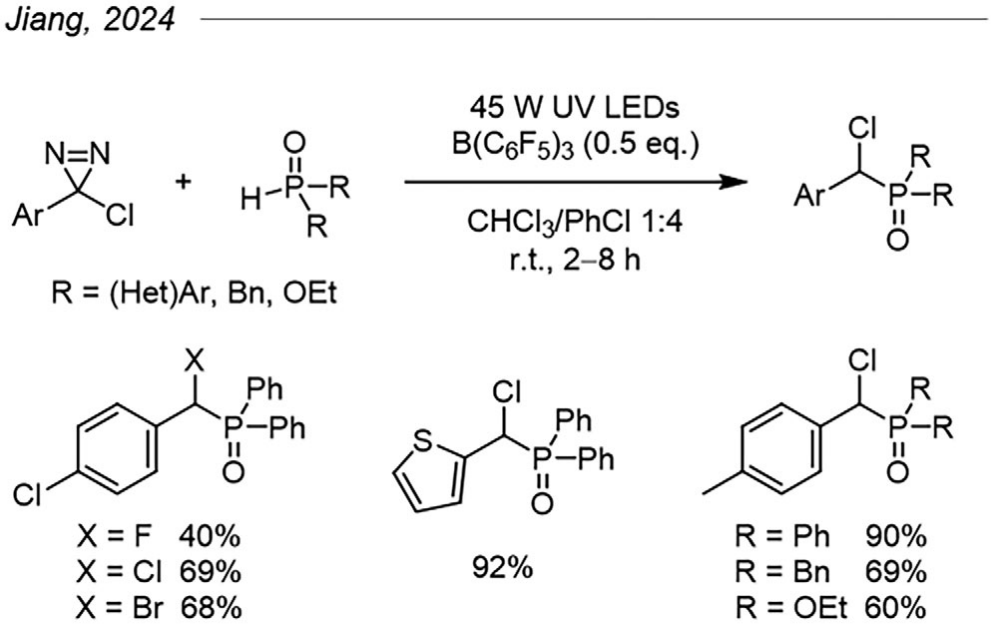
P─H insertion of halodiazirines into *H*‐phosphorous oxides, along with a few examples.

Beyond their potential for cycloadditions with unsaturated compounds, chlorodiazirines were shown to generate α‐chlorocarbenes under UV‐light irradiation that smoothly inserted into P─H bonds of *H*‐phosphorous oxides (Figure 20).^[^ ^80^ ^]^ The reaction proceeded in good to excellent yields and tolerated a diversity of aryl substituents on the *H*‐phosphorous oxide. Based on two additional examples, bromodiazirines performed similarly, whereas fluorodiazirines afforded a slightly decreased yield compared to the chlorodiazirine counterpart. Mechanistic investigations unveiled triplet and/or radical intermediates that the authors presumed descend from the triplet carbene.

### 5.8 Access to *gem*‐Difluoroalkenes and Tetrafluorocyclopropanes

**Figure 21:**
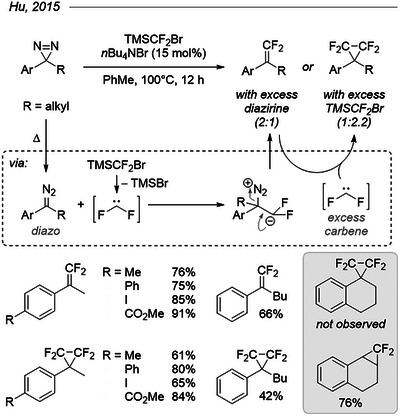
Difluoroolefination of aryl alkyl diazirines and subsequent access to tetrafluorocyclopropanes with excess difluorocarbene.

Jinbo Hu and his group formally “coupled” two carbenes to access certain 1,1‐difluoro‐2‐arylalkenes that could not be prepared from the corresponding diazoalkanes.^[^ ^81^ ^]^ Simultaneous generation of difluorocarbene from TMSCF_2_Br and thermal isomerization of aryl alkyl diazirines to their diazo isomer at 100 °C in toluene afforded the 1,1‐difluoroalkenes in good yields when diazirine was used in a twofold excess (Figure 21). Of course, the produced alkenes being susceptible to cyclopropanation with excess difluorocarbene, the reaction yielded the corresponding tetrafluorocyclopropanes when diazirines were used as limiting reagents. Interestingly, no 1,2‐rearrangement occurred in acyclic alkyl chains, but reaction of tetralone‐derived diazirine with an excess of difluorocarbene did not afford the expected product: instead, a fused difluorocyclopropane reasonably arising from cyclopropanation of the 1,2‐rearrangement product (1,2‐dihydronaphthalene) was obtained.

### 5.9 Skeletal Editing via a Carbynyl Cation Equivalent

**Figure 22:**
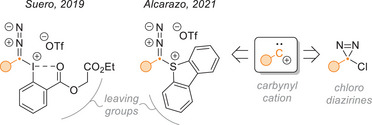
Examples of carbynyl cation equivalents and comparison with chlorodiazirines.

**Figure 23:**
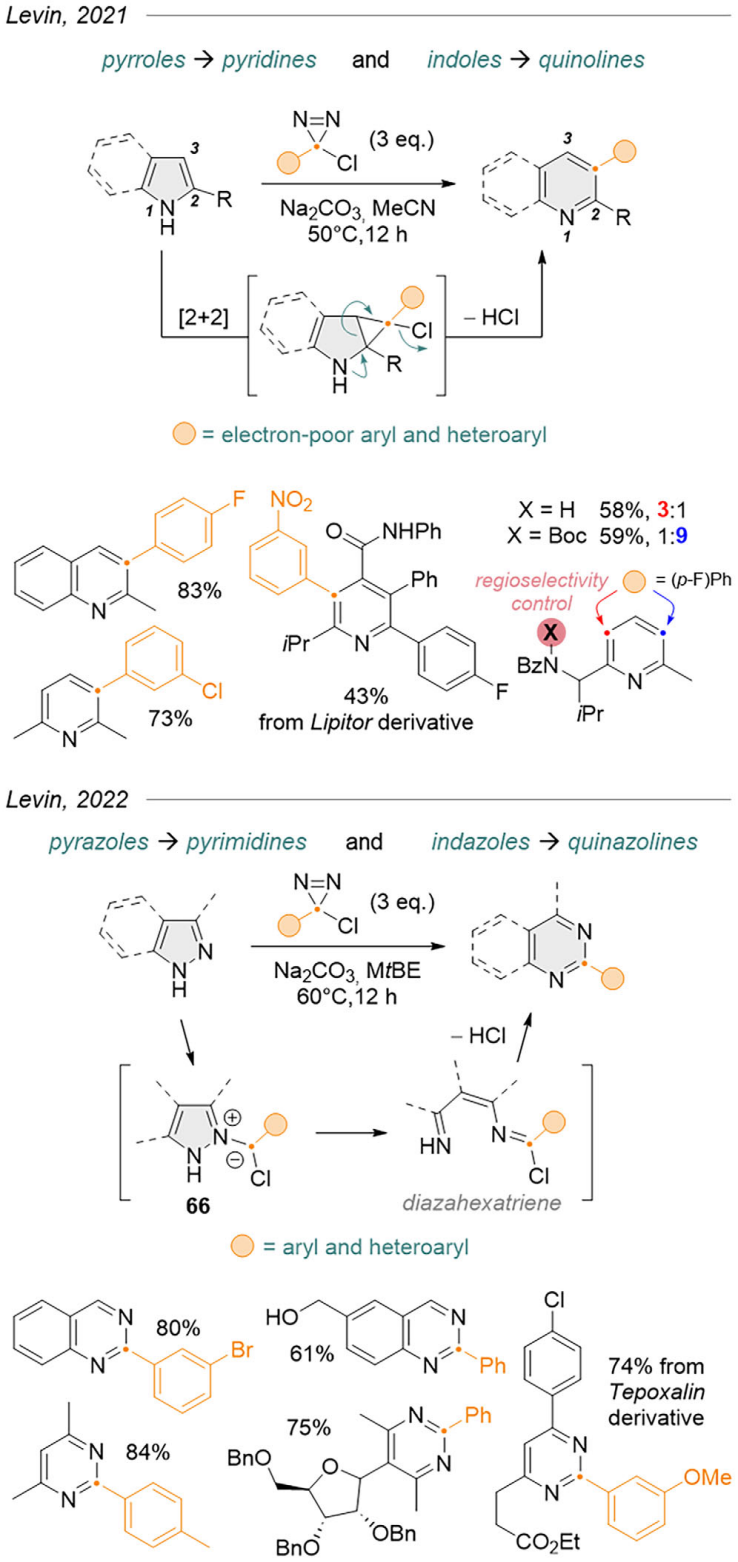
Formal (hetero)aryl methide insertion into aromatic C─C or N─N bonds via thermal decomposition of arylchlorodiazirines.

Taking inspiration from the Ciamician‐Dennstedt rearrangement and the work of Suero et al. on carbynoids (Figure 22),^[^ ^82^, ^83^, ^84^ ^]^ Levin et al. used chloroaryldiazirines as carbynyl cation equivalents in the skeletal editing of indoles and pyrroles.^[^ ^15^ ^]^ Using the extensive pool of commercial (hetero)aryl‐amidines, which they converted in one step to the corresponding chlorodiazirines via the Graham reaction (*vide supra*), they were able to achieve regioselective insertions of formal aryl‐methides into aromatic C─C bonds (Figure 23, top). Electron‐rich 2‐substituted indoles and pyrroles proved very effective substrates, whereas electron‐poor derivatives or those lacking a substituent in the 2‐position would provide little or none of the desired products. Unsymmetrical pyrroles gave mixtures of regioisomers in varying ratios, but insertion into trisubstituted substrates proceeded smoothly on the less hindered side, suggesting the dominance of steric over electronic effects. The diazirine coupling partners comprise arenes and heteroarenes with a broad range of electron‐withdrawing substituents, although methoxy also worked well when in the *meta* position. Electron‐rich chlorodiazirines afforded exclusively the corresponding aldehydes, and alkylchlorodiazirines underwent typical rearrangements to vinyl chlorides and azines.

They soon extended the concept to the skeletal editing of pyrazoles and indazoles toward pyrimidines and quinazolines, respectively (Figure 23, bottom).^[^ ^85^ ^]^ Despite the similarity of the reaction conditions, experimental results and DFT calculations support a different mechanism where the heteroaromatics’ N─N bond of intermediate ylide **66** is first cleaved to afford an open 1,5‐diazahexatriene that then ring‐closes with concomitant loss of chloride. The use of methyl *tert*‐butyl ether as a solvent proved critical in obtaining satisfying yields for these substrates. Additionally, the reaction could be performed with free alcohols, esters, and a few other functionalities not tolerated on pyrroles/indoles.

**Figure 24:**
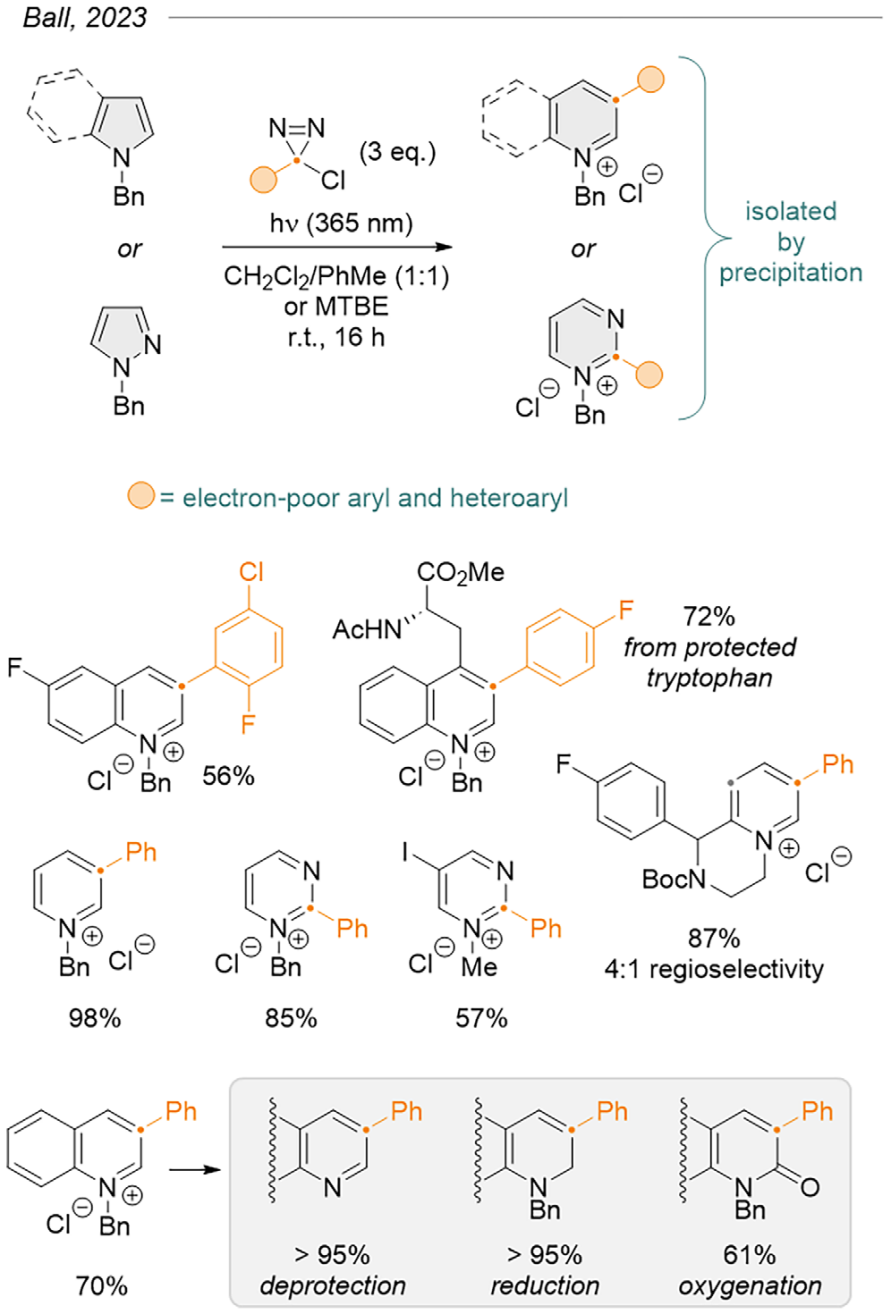
Formal (hetero)aryl methide insertion into aromatic C─C or N─N bonds via photochemical decomposition of arylchlorodiazirines. The azinium salts can easily be further derivatized.^[^ ^50^ ^]^

Quickly thereafter, Ball et al. improved upon Levin's approach by activating *N*‐protected chlorodiazirines under photochemical conditions (Figure 24).^[^ ^50^ ^]^ By installing an *N*‐substituent, the reaction produced *N*‐heterocyclic cations that spontaneously precipitated as chloride salts in apolar solvents. This purposeful choice was meant to remove from the reaction medium both the chloride anion, which was identified as a poison of the reaction, and the product, to avoid its degradation with the excess carbene. Besides, the photochemical activation could be performed at or below room temperature. The approach proved tolerant to a wide range of substituents, including electron‐withdrawing ones, and lifted the previous requirement for a substituent at the 2‐position. Both of these are remarkable improvements to previous reports. Benzyl was determined as a far superior *N*‐substituent during reaction optimization, although a few examples displaying phenyl, methyl, or alkyl groups were reported. On quinolinium products, the *N*‐benzyl substituent ended up activating the *N*‐heterocyclic salt toward further derivatization via deprotection, oxygenation, or various (regio)selective reductions.

**Figure 25:**
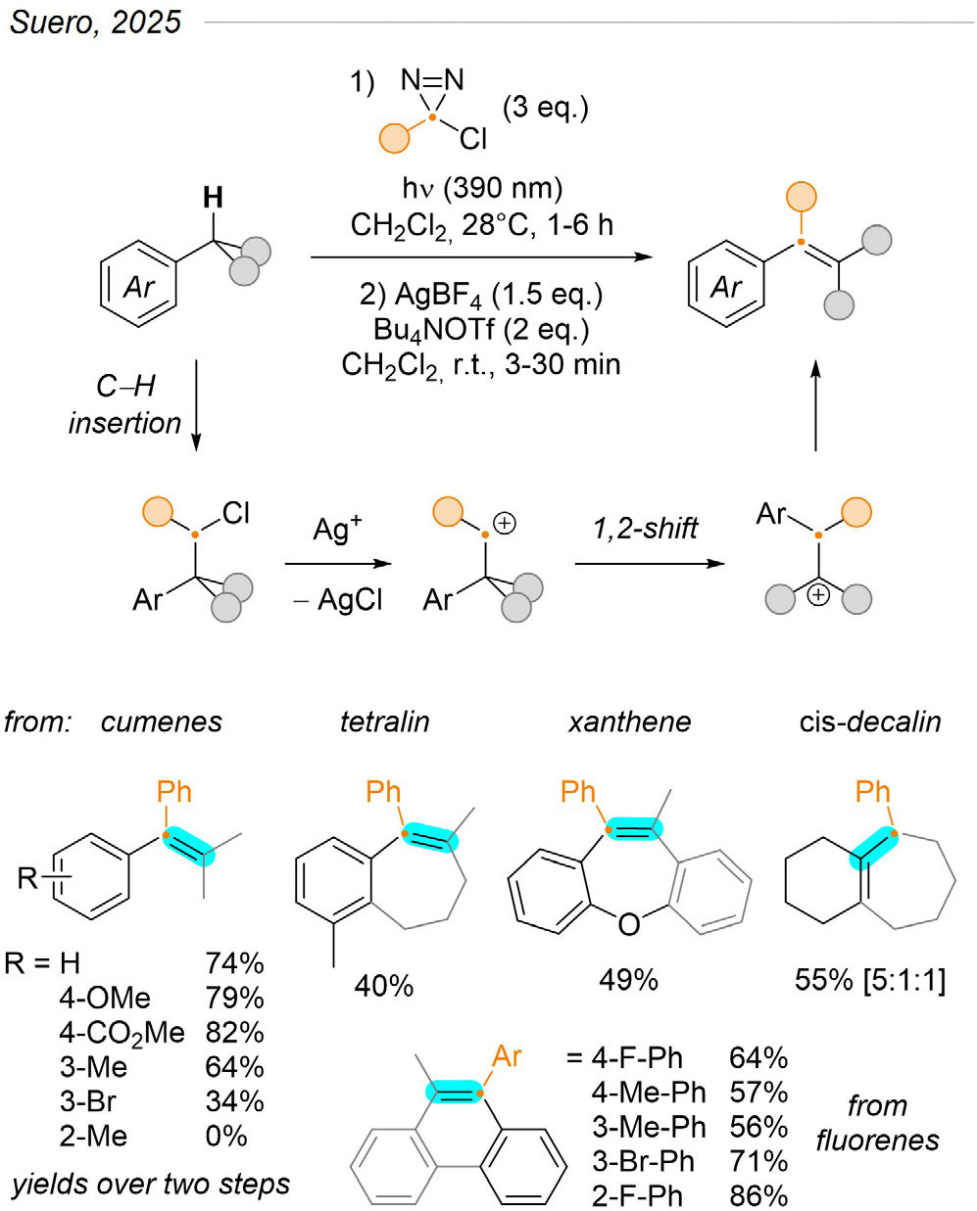
Insertion of aryl methide into single C─C bonds via a two‐step process.^[^ ^86^ ^]^

Finally, similar conditions were used by Suero et al. to effect an aryl methide insertion in single C─C bonds, affording tetrasubstituted alkenes (Figure 25).^[^ ^86^ ^]^ This skeletal‐editing transformation involved two steps: first, photolysis of aryl chlorodiazirines produced carbenes that inserted preferentially in a tertiary C─H bond, and the intermediate benzyl chlorides underwent a silver(I)‐promoted Wagner‐Meerwein rearrangement leading to the alkene product. Many substituents were tolerated on the aromatic ring, although not in the *ortho* position, likely due to a steric clash with the approaching carbene during C─H insertion. Yields for this insertion were particularly high with dibenzylic C─H bonds (xanthenes, fluorenes), but were in some cases moderated by modest yields and/or selectivity in the cationic rearrangement. The diazirine scope included electron‐poor to neutral aromatics. Of note, the transformation was applied successfully on *cis*‐decalin, showing compatibility with all‐alkyl substrates at the expense of selectivity.

### 5.10 Vicinal Bis‐Diazirines as Alkyne Precursors

**Figure 26:**
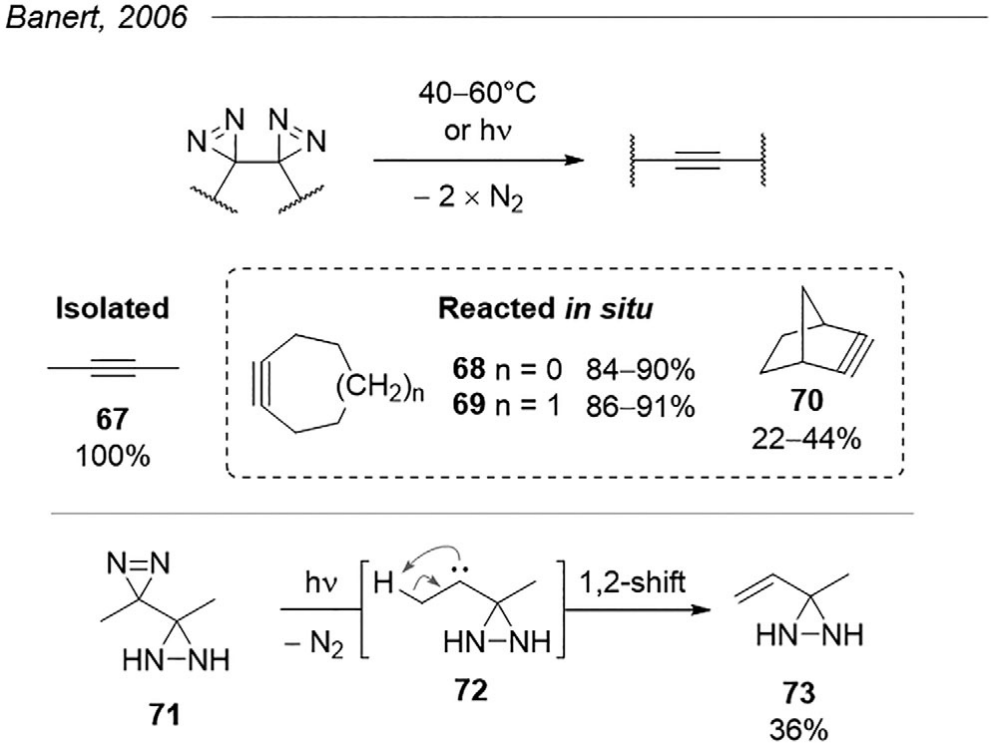
Top: The decomposition of vicinal bis‐diazirines leads to alkynes, even in strained systems. (Yield for **67** is isolated; yields for **68–70** are for the trapped species.) Bottom: Photolysis of mono‐diazirine control **71** yields the rearranged alkene, suggesting an intermediate carbene.

The unusual yet particularly interesting structure of vicinal bis‐diazirines was reported by Banert et al. in 2006.^[^ ^87^ ^]^ Using standard routes (*vide supra*) from vicinal imines, they were able to isolate four derivatives bearing this peculiar motif. All decomposed slowly at room temperature but could be kept intact at −30 °C for long periods. Mild heating (40‐60 °C) or photolysis (wavelength not mentioned, but probably around 350 nm) triggered the release of molecular nitrogen and the formation of the corresponding alkynes (Figure 26, top). But‐2‐yne **67** was isolated in quantitative yield, and no intermediate was observed under either set of conditions, suggesting a potential cooperative mechanism bypassing reactive free carbenes. Interestingly, the semi‐oxidized species **71** did form the mono‐carbene species upon irradiation, as evidenced by the isolation of the 1,2‐rearrangement product **73** (Figure 26, bottom). Strained alkynes **68** and **69** were trapped through various cycloadditions in excellent yields, thereby confirming the excellent conversion, whereas fused bicyclic norbornyne **70** proved harder to generate and/or trap.

## 6 Diazirines as Electrophilic Nitrogen Donors

**Figure 27:**
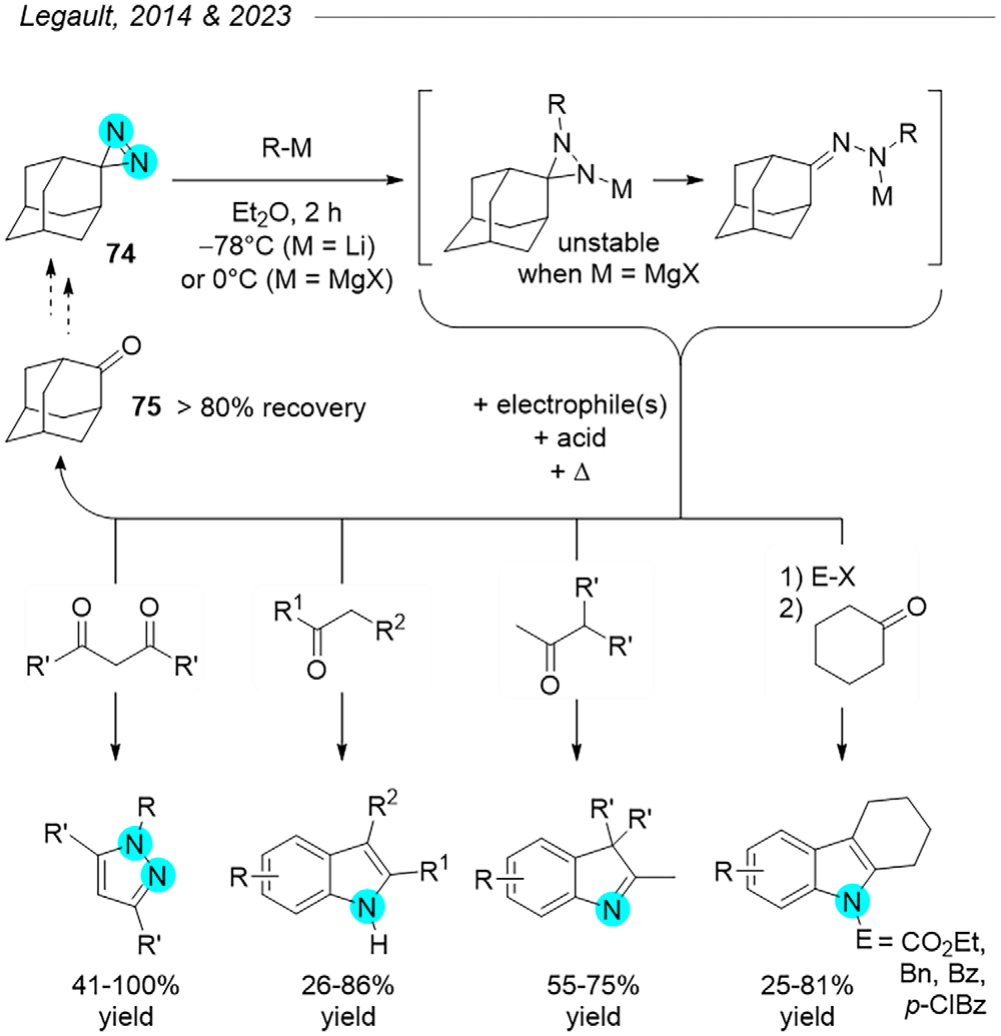
3,3‐Adamantyldiazirine **74** is used as an electrophilic nitrogen donor, where organo‐lithium and ‐magnesium reagents add across the N═N double bond to yield the corresponding metalated diaziridines and/or hydrazones. Those can be condensed in situ with 1,3‐diketones to afford *N‐*substituted pyrazoles or enolizable ketones to produce a variety of indoles. The by‐product adamantanone **75** is recovered and recycled.

Whereas Schmitz and coworkers realized the electrophilic potential of diazirines as early as 1961,^[^ ^88^ ^]^ it remained mostly unexploited for > 50 years.^[^ ^89^ ^]^ In 2014, Legault^[^ ^90^ ^]^ et al. revisited this character and used adamantyl‐diazirine **74** as a nitrogen donor for the preparation of *N*‐substituted diaziridines and hydrazones, in turn leading to pyrazoles (Figure 27). The addition of organometallic reagents across the N═N double bond proceeded quickly and in quantitative yields, although the stability of the resulting *N*‐substituted diaziridine strongly depended on the metal. Indeed, phenyl Grignard rearranged in situ to the corresponding hydrazone, a process which was slowed at −40 °C, whereas phenyl lithium at −78 °C afforded the metalated diaziridine. A broad range of lithium and magnesium nucleophiles were tolerated, including alkyl, allyl, benzyl, and a variety of substituted aryl organometallic reagents. Further reaction of either the diaziridines or hydrazones with 1,3‐diketones yielded trisubstituted pyrazoles in excellent yields, along with adamantone **75** (> 80% recovery). The latter could be recycled to the starting adamantyl diazirine **74** in only two steps with inexpensive reagents. Taking advantage of the hydrazines produced in situ from the acid‐promoted hydrolysis of adamantyl hydrazones, the methodology was expanded in 2023 to produce indoles with a variety of substituents via the Fischer process.^[^ ^91^ ^]^

**Figure 28:**
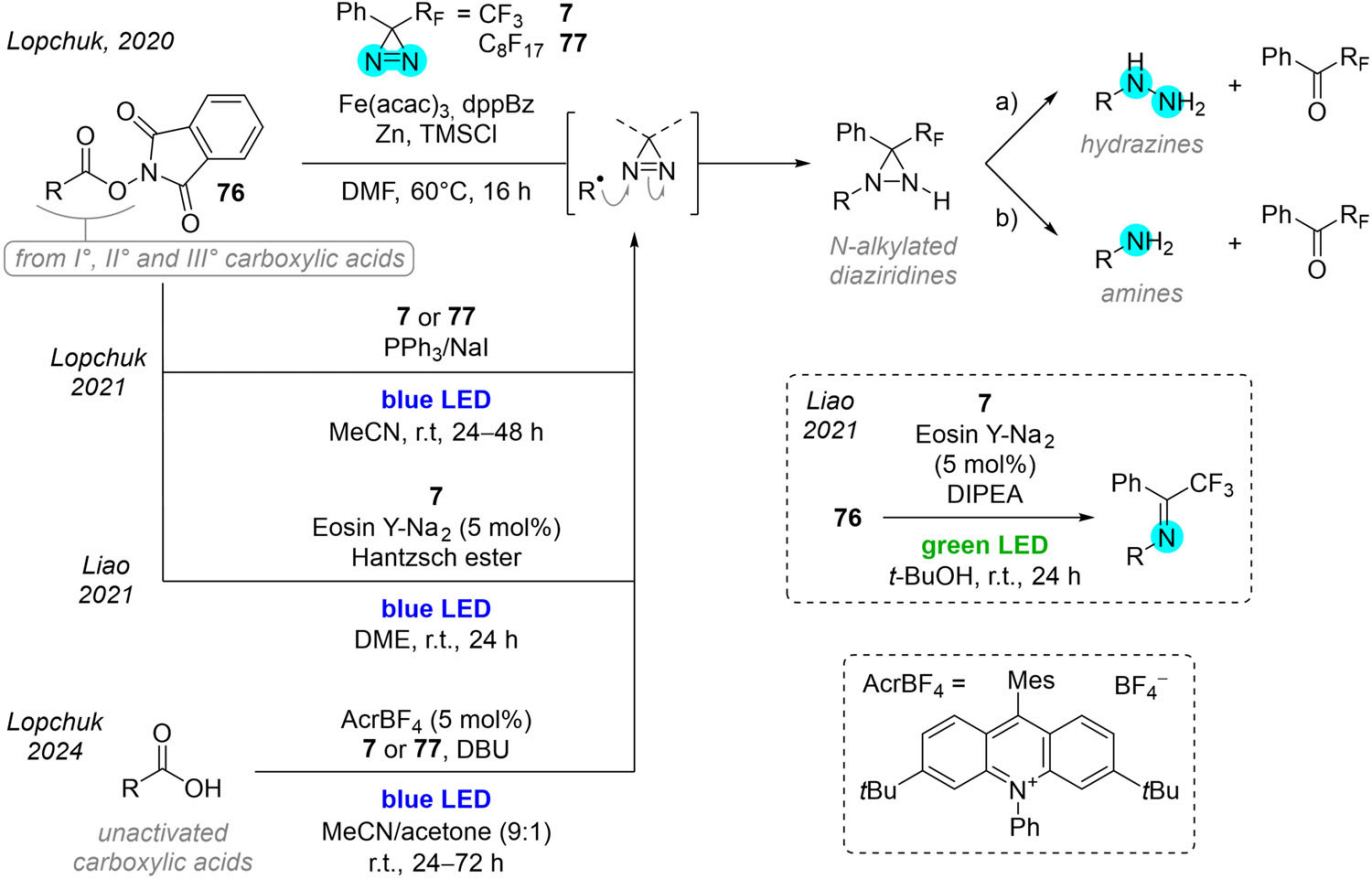
Decarboxylative amination strategy using fluorinated diazirines **7** or **77** as electrophilic nitrogen donors,^[^ ^95^, ^96^, ^97^, ^98^ ^]^ where the intermediate *N*‐alkylated diaziridines are converted selectively to a hydrazine or an amine. a) MsOH (or H_2_SO_4_ or *p*‐TsOH), EtOH, 90 °C, 16 hours; b) HI (or LiCl/TMSCl or HCl/I_2_ or HCl/NaI), MeCN, r.t., 2 hours.

Radicals can also add across the N═N double bond of diazirines to forge new C─N bonds. Early but sparse reports from Krespan^[^ ^92^ ^]^ and Barton^[^ ^93^, ^94^ ^]^ prompted Lopchuk et al. to study aryl polyfluorinated diazirines as electrophilic nitrogen donors to redox‐active esters (i.e.*, N*‐(acyloxy)phthalimides, Figure 28).^[^ ^95^ ^]^ Upon redox‐promoted decarboxylation, the generated radicals reacted with Brunner's trifluoromethyl phenyl diazirine **7** (1.5 to 3 equiv.) to afford diaziridines, which could be further derivatized to the corresponding amines or hydrazines through selective hydrolysis. It is remarkable that the diazirine was not simply reduced in the reaction conditions, contrary to the diazines tested in control experiments. The sheer number of carboxylic acid precursors (> 50 examples), scaled‐up and one‐pot procedures reported by the authors speak to the robustness of the process. For primary redox‐active esters, the use of perfluorooctyl‐phenyldiazirine **77** proved more efficient, although the authors did not provide a rationale or justification. The perfluoroalkyl tag also enabled fluorous solid‐phase extraction, which was exemplified by some expeditious purifications of crude mixtures after either step. An improved, metal‐free photodecarboxylation under blue‐light irradiation provided higher yields for primary esters even with Brunner's TPD **7**.^[^ ^96^ ^]^ Liao et al. independently reported photocatalytic redox conditions with Eosin Y with similar outcomes,^[^ ^97^ ^]^ with the interesting observation that irradiation with green light in the presence of DIPEA in *t*‐BuOH allowed them to isolate the phenyl trifluoromethyl imines *en route* to amines. More recently, the Lopchuk group used an acridinium photocatalyst to achieve the direct, metal‐free catalytic photodecarboxylative amination of unactivated carboxylic acids, thereby avoiding the need for an added reductant.^[^ ^98^ ^]^

**Figure 29:**
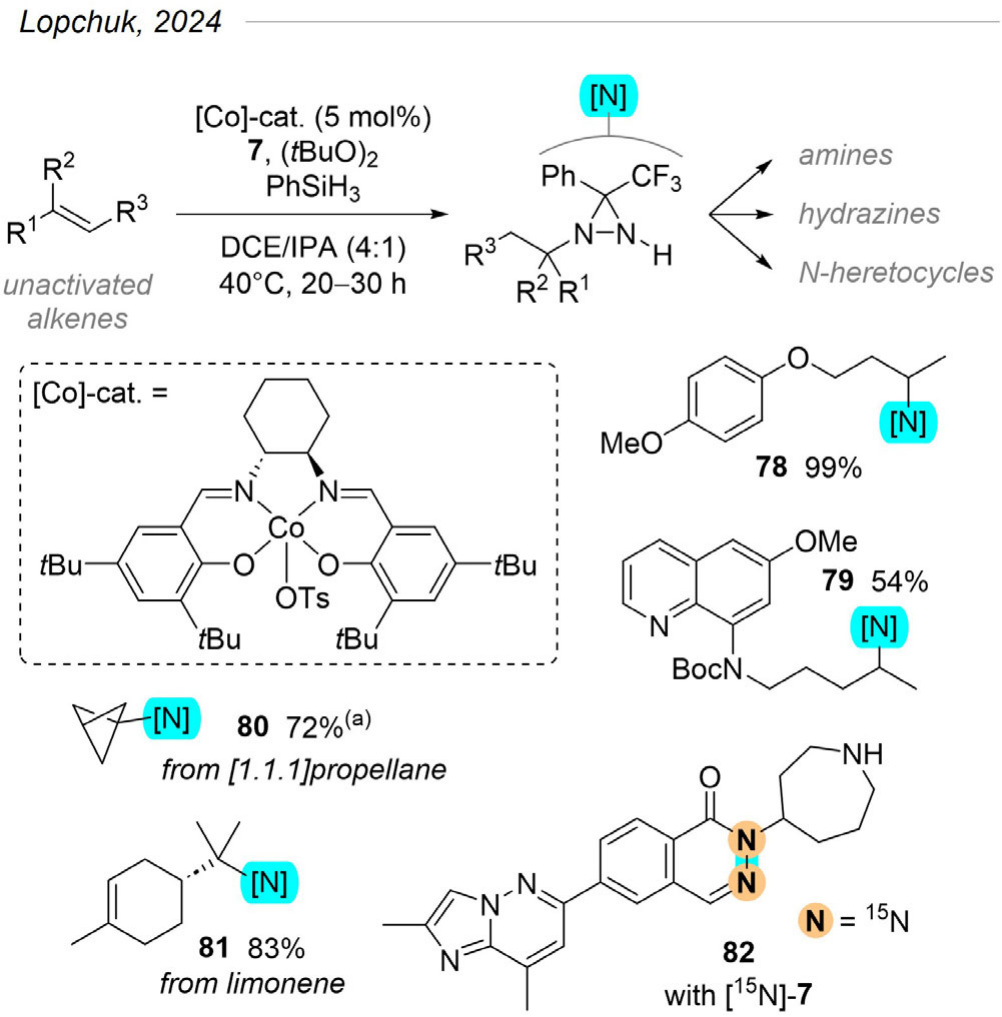
Regioselective hydroamination of unactivated alkenes using diazirine **7** as a nitrogen donor.^[^ ^99^ ^]^ (The obtained *N*‐alkylated diaziridines are converted selectively to amines, hydrazines, or *N*‐heterocycles. A few representative examples of products are given.) ^(a)^ [1.1.1]propellane reacted best with Mn(dpm)_3_ as a catalyst.

Leveraging their acquired expertise in using polyfluorinated diazirines as radical acceptors, Lopchuk et al. elected to tackle hydroamination of unactivated alkenes.^[^ ^99^ ^]^ Doing so, they aimed to associate the wide availability and diversity of olefins with the user‐friendliness of diazirine reagents as single or double nitrogen donors. In their optimization phase, they identified di‐*tert*‐butyl peroxide as the crucial additive allowing high yields with a cobalt‐salen catalyst (Figure 29). Here again, the large number (> 50) of substrates tested nicely illustrates the tolerance of this strategy to a very broad range of functional and protecting groups, as well as the applicability to tri‐substituted, cyclic, and/or sterically encumbered motifs. Downstream conversions of a select few diaziridine products to amines, hydrazines, or heterocycles exemplify the generality of the approach and justify its addition to chemists’ toolbox. Importantly, the authors also described the preparation and use of [^15^N]‐**7** to access ^15^N‐labeled **82**, an RNA splicing candidate. As exemplified in the next section below, the ^15^N‐labeling of compounds of biological interest is an important analytical tool due to the high signal‐to‐noise ratio detected in NMR, MS, and MRI.

## 7 Diazirines as NMR Probes: SABRE‐SHEATH

**Figure 30:**
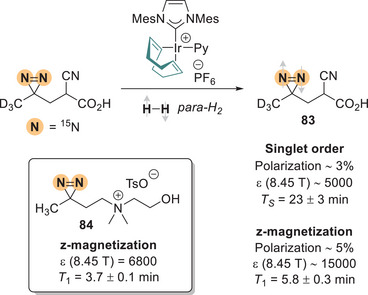
Signal Amplification By Reversible Exchange (SABRE) in SHield Enables Alignment Transfer to Heteronuclei (‐SHEATH).

The technique of Signal Amplification by Reversible Exchange (SABRE) improves NMR sensitivity by hyperpolarizing spins in the molecule(s) of interest, allowing detection of smaller concentrations of analytes. It is therefore interesting, in the context of MRI, for example, to study cellular processes that are otherwise overshadowed by the sheer amount of water (and its protons) present in all biological systems. It relies on the reversible binding of *para*‐hydrogen (as a polarization source) and the target molecule to a catalyst (typically iridium‐based), where polarization transfer occurs through *J*‐couplings in milli‐Tesla fields (Figure 30). When performed in particular —yet inexpensive— micro‐Tesla equipment, SABRE in SHield Enables Alignment Transfer to Heteronuclei (SABRE‐SHEATH), which is a further development of the technique where low‐γ nuclei like ^15^N are hyperpolarized directly rather than protons. After only bubbling *para*‐H_2_ in the sample for 5 minutes, the signal enhancement can reach four orders of magnitude, while the relaxation times are dramatically increased.^[^ ^100^ ^]^

^15^N_2_‐Diazirines are excellent probes for this approach since they are very stable at room temperature and resistant to nucleophiles, acids, and bases. Their small size and their generally low impact on biological properties have already been extensively demonstrated in PAL. Besides those perks, Warren et al. recognized the added benefit of having two adjacent ^15^N, whose strong coupling led to a long‐lived singlet state.^[^ ^16^ ^]^ On top of the regular z‐magnetization (15,000‐fold signal enhancement), the singlet order of **83** corresponds to an anti‐aligned spin state (Figure 30, right side of the arrow), which exhibits dramatically increased lifetimes (*T*_S_ = 23 minutes), allowing hour‐long tracking of the hyperpolarized molecule. This relaxation‐protected spin order state allows storage of polarization, which can then be transferred on demand to neighboring ^1^H nuclei, a method of particular interest in the context of hyperpolarized contrast agents.^[^ ^101^ ^]^ The diazirine motif itself ensures binding to the iridium polarization transfer catalyst, with no other features required.^[^ ^102^ ^]^ The hyperpolarization of choline‐derivative **84** by SABRE‐SHEATH “demonstrate[s] the applicability of ^15^N_2_‐tags within biologically relevant molecules.” Yet the poor solubility of polarizing partners in water is an important limitation of the technique. Nonetheless, ^15^N_2_‐diazirines can alternatively be used in dissolution dynamic nuclear polarization in D_2_O, a more expensive method that yields similar relaxation time but much higher signal enhancements.^[^ ^103^ ^]^

## 8 Diazirines in Materials Science

All along the development of diazirine chemistry, the capacity to create covalent bonds between two different substances has inspired many groups to develop applications in materials science. Although an exhaustive inventory of this field is not in the scope of this review, we want to highlight here a diversity of approaches where diazirine‐based reagents enable the creation, functionalization, and/or crosslinking of various materials.

### 8.1 Polymers Made From Diazirines

**Figure 31:**
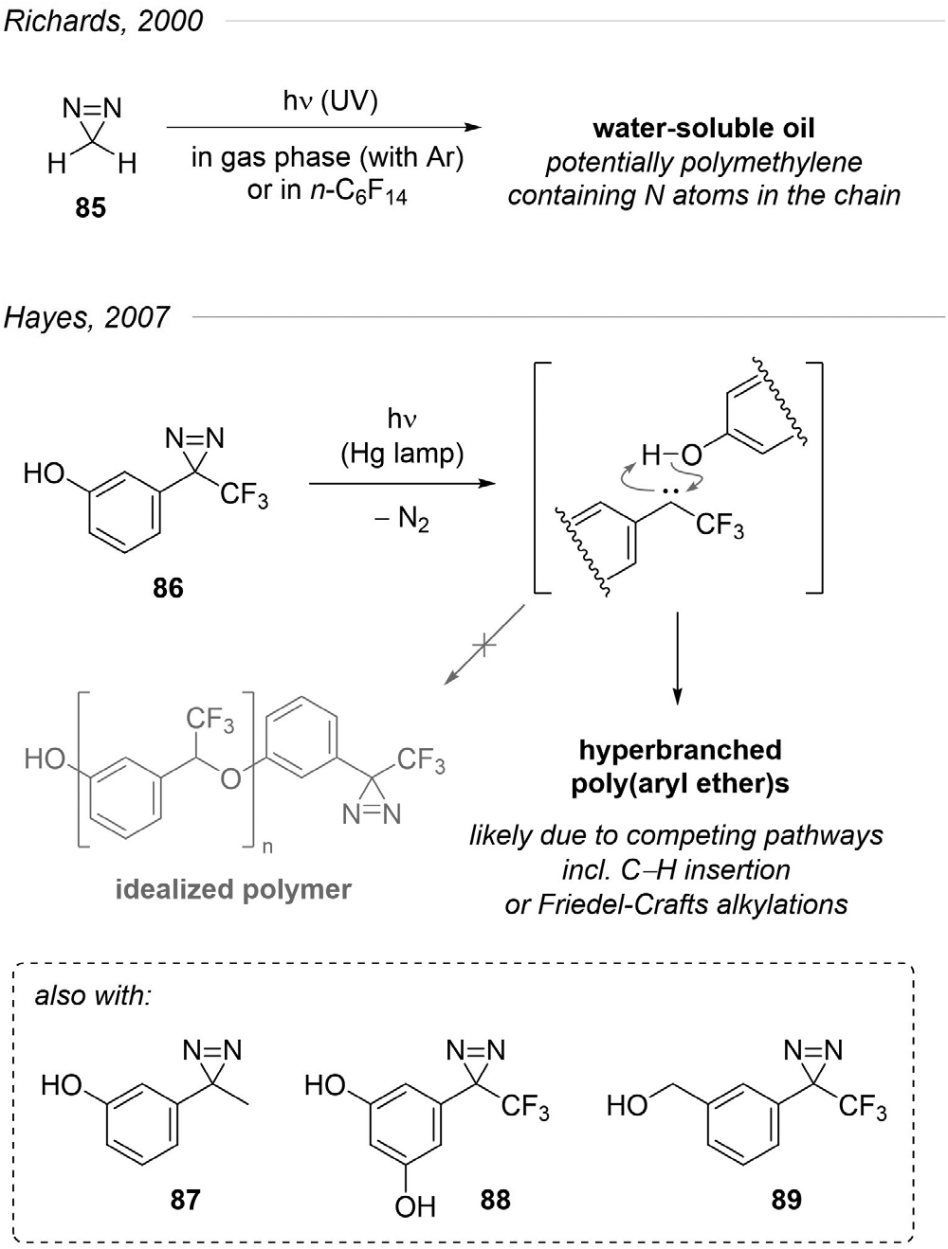
Rare reports of ill‐defined polymers made from diazirines.

In the course of their study of methylene (:CH_2_) generated by photolysis of 3*H*‐diazirine **85**, Richards et al. produced a water‐soluble oil that they attempted to characterize (Figure 31).^[^ ^104^ ^]^ They were not able to determine a definite structure but posited the material to be polymethylene (linear or branched) incorporating nitrogen atoms. An important piece of data leading to this conclusion was the significant change in the mass spectrum of the oil when produced from [^15^N]‐**85**.

A few years later, Hayes et al. proposed a rational synthesis of poly(aryl ether)s via homopolymerization of hydroxy‐functionalized diazirines.^[^ ^105^ ^]^ In their approach, the direct photolysis (UV) of the diazirine monomers **86–89** would produce carbenes that should easily insert into the O─H bonds of another monomer or at the tail‐end of a growing polymer chain. In practice, however, pathways competing with the sought O─H insertion (e.g., Friedel‐Crafts alkylations)^[^ ^106^ ^]^ promoted the formation of hyperbranched poly(aryl ether)s whose properties (solubility, molecular weight, etc.) heavily depended on the monomer and solvent employed.

### 8.2 Functionalization of Surfaces and Materials

**Figure 32:**
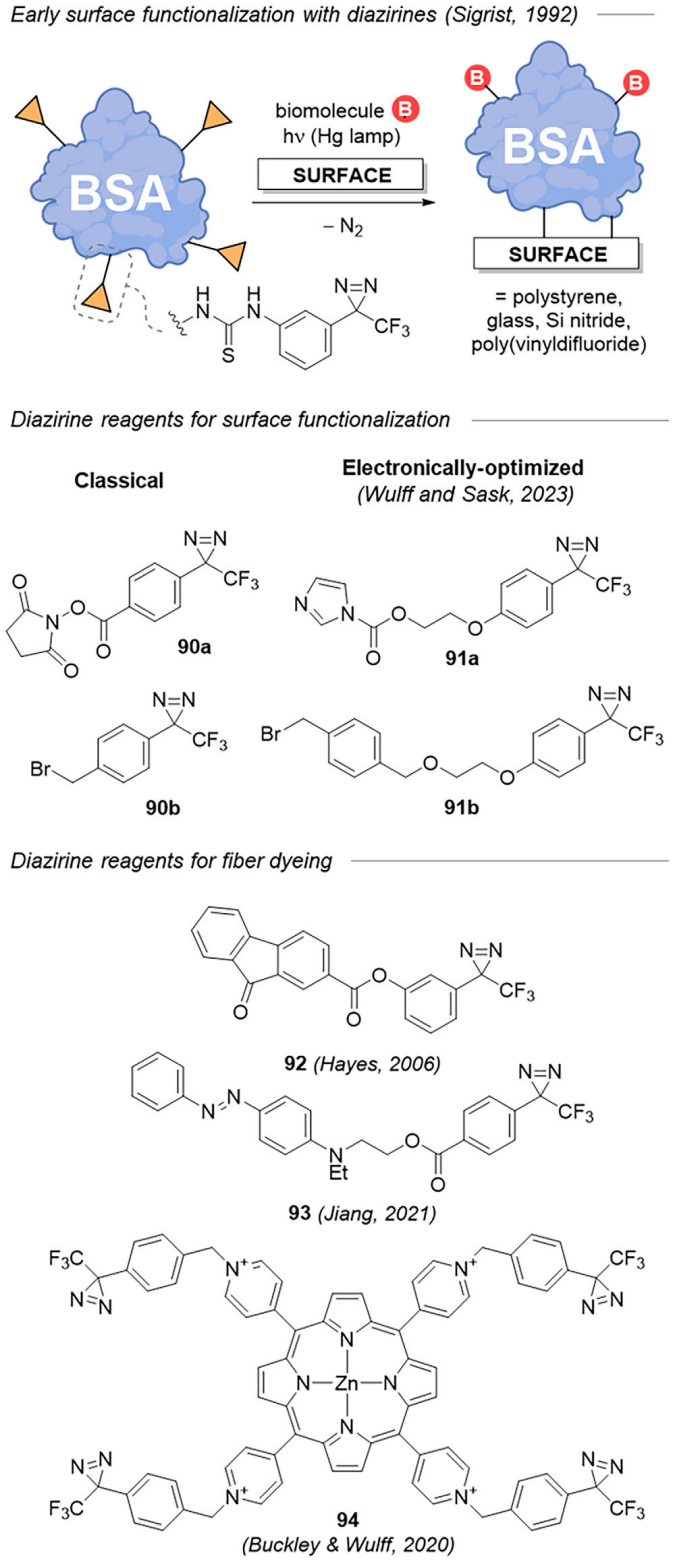
Examples of diazirine conjugates used for the functionalization of surfaces and materials.

In an early, direct translation of PAL to materials science, Sigrist et al. used a bovine serum albumin (BSA) bearing multiple TAD warheads for the covalent immobilization of several biomolecules on inert surfaces such as polystyrene, glass, silicon nitride, or even poly(vinyl difluoride) (Figure 32, top).^[^ ^107^, ^108^ ^]^ From a more global point of view, the functionalization of surfaces with diazirine reagents enables the “reactivity reprogramming” of a given surface by installing more reactive moieties than those originally present. Bifunctional electrophilic diazirines like those presented in Figure 32 are particularly appropriate to render any surface reactive toward biomolecules, which are typically rich in nucleophilic residues (e.g., lysines, serines, and cysteines in proteins). Classical TADs equipped with an activated ester (**90a**) or benzyl bromide (**90b**) are widespread in the literature,^[^ ^109^, ^110^, ^111^ ^]^ but recently the Sask and Wulff groups implemented electronically optimized diazirine moieties **91a‐b** in the functionalization of PDMS.^[^ ^112^ ^]^ The Wulff group also employed the mirror approach with diazirine‐armed poly(ethylene imine) primers (not shown here) to covalently attach highly nucleophilic amines at the surface of low surface energy materials (e.g., polyethylene), thereby dramatically increasing their bonding with common electrophilic adhesives (e.g., epoxy resins) or with electrophilic dyes.^[^ ^113^, ^114^ ^]^ The direct covalent attachment of chromophores and dyes to synthetic fibers (nylon‐6,6 or polypropylene) was easily performed with bespoke diazirine reagents such as **92,93**.^[^ ^115^, ^116^ ^]^ Harnessing this strategy, Buckley, Wulff et al. were able to functionalize commodity polyolefin or polyester with a photosensitizer **94** (zinc porphyrin), enabling photodynamic pathogen inactivation, for example, on melt‐blown polypropylene masks used during the 2020 pandemic.^[^ ^117^, ^118^, ^119^ ^]^

**Figure 33:**
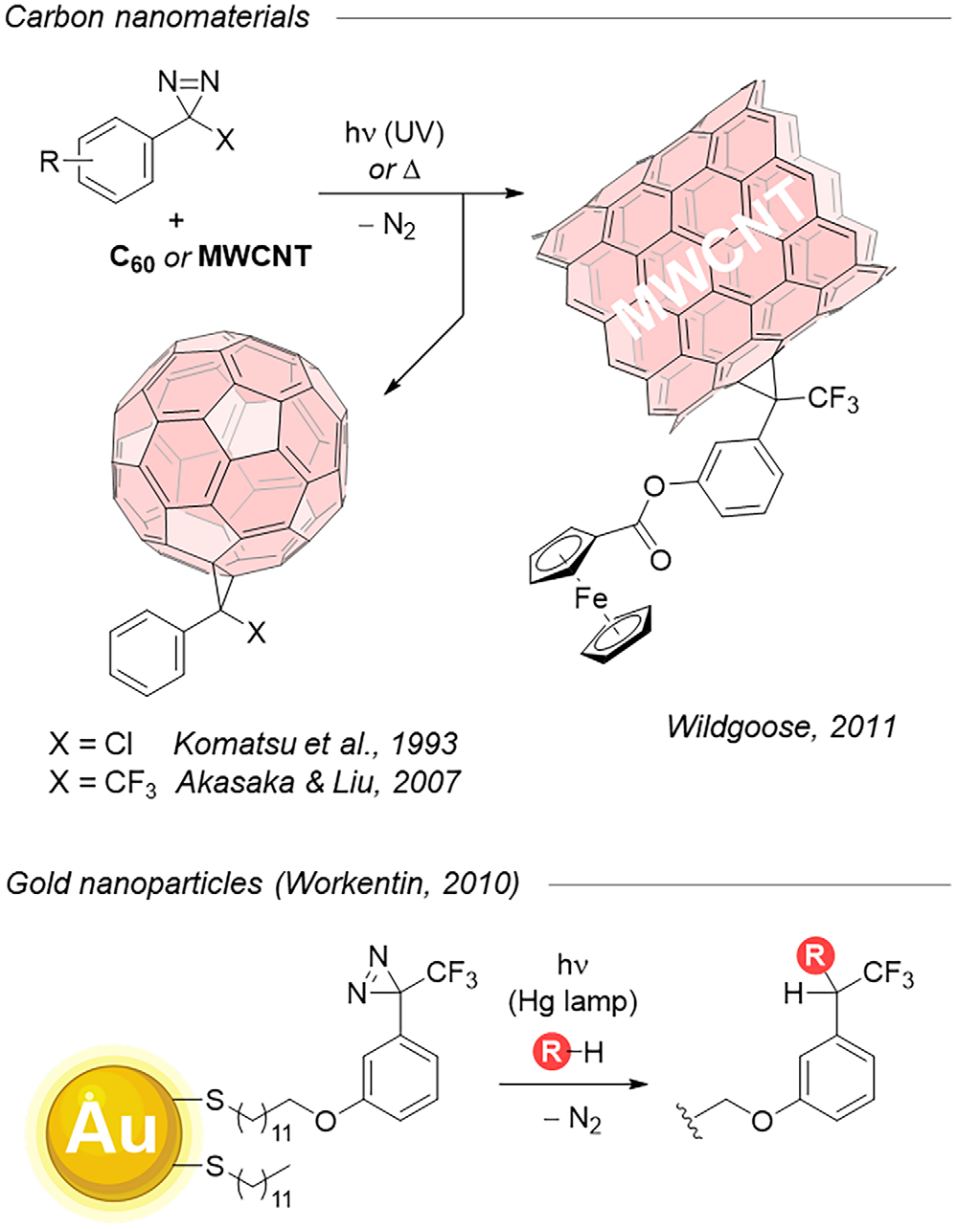
Modification of nanomaterials with diazirines.

Nanomaterials have also been modified with diazirines. Diazirines were used to directly functionalize carbon materials such as fullerene^[^ ^120^, ^121^, ^122^ ^]^ or nanotubes,^[^ ^55^ ^]^ taking advantage of the efficient reaction between diazirine‐born carbenes and π‐rich surfaces (Figure 33, top). In a mirror approach, gold nanoparticles were decorated with TAD motifs (in their stabilizing lipophilic monolayer) that were subsequently used to capture small molecules via O─H or N─H insertions (acids, alcohols, amines) or even cyclopropanation (with methyl acrylate or styrene, not shown here for clarity).^[^ ^123^ ^]^ This allowed Workentin et al. to quickly access a structural diversity in the interfacial layer of their nanoparticles (Figure 33, bottom). A similar diazirine‐armed, alkoxy‐tethered ferrocene conjugate was used by the same group for on‐demand functionalization of a glassy carbon surface (not shown for clarity).^[^ ^124^ ^]^

### 8.3 Multivalent Diazirines as Universal Crosslinkers

**Figure 34:**
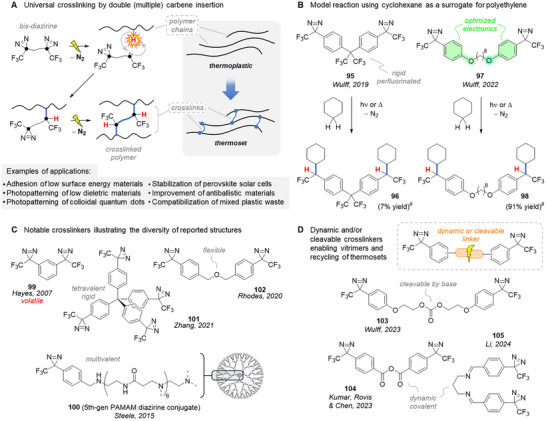
Crosslinking polymers with bis‐diazirine via successive insertions of carbenes. ^#^ = thermal activation at 140 °C in cyclohexane.

When using diazirines in PAL, the term “crosslinking” is often used somewhat incorrectly since it normally should refer to the attachment of at least *two* polymer chains together. In fact, to perform *actual* crosslinking, a multivalent diazirine (valency ≥ 2) is required. The idea of leveraging diazirines as stable carbene precursors to crosslink polymers arose in the 2000s, with a few isolated (and important) communications reporting preliminary results around this concept.^[^ ^14^, ^125^, ^126^ ^]^ Yet it is a 2019 seminal article by Wulff et al. that really gave this approach a wide visibility by demonstrating the broad application of simple bis‐diazirines as universal crosslinkers for aliphatic polymers (Figure 34A).^[^ ^12^, ^127^ ^]^ The successive decomposition of each diazirine moiety into a carbene that undergoes insertion reactions with the surrounding bonds is at the center of this strategy that enabled many and very diverse applications ranging from adhesion to the photopatterning of low‐dielectric materials in microelectronics. The design, exploitation, and rational improvements of diazirine‐based universal polymer crosslinkers have very recently been the subject of a detailed account by Wulff et al.,^[^ ^13^ ^]^ and here we only want to give a brief overview of this fast‐expanding field.

Structurally, bis‐diazirine crosslinkers simply connect two (or more) trifluoromethyl phenyl diazirine (Brunner's TPD **7**, Figure 7) through a linker, which proved a critical parameter driving most of the successive improvements in the field. In particular, a sufficient molecular weight of a crosslinker is paramount to reduce its volatility and explosivity. These concerns prompted Wulff et al. to build their first usable crosslinker, **95** (Figure 34B), around a rigid perfluorinated isopropylene tether, which was meant to avoid both intra‐ and intermolecular reactions of the crosslinker with itself. The ability of **95** to undergo a double C─H insertion under both thermal and photochemical activation was thoroughly demonstrated in cyclohexane, used as a small‐molecule surrogate for polyethylene.^[^ ^12^ ^]^ The efficiency of this model reaction (measured as the isolated yield of bis‐cyclohexane adducts) was later dramatically improved (10‐fold) by building **97** around a bis‐alkoxy linker that stabilizes the singlet state carbene (see *Carbene Generation* above).^[^ ^128^ ^]^

Multivalent diazirine crosslinkers can thus crosslink any material that presents X─H bonds (X = C, N, O, etc.), making them applicable to virtually any aliphatic polymer: polyethylene, polypropylene, silicone, polycaprolactone, poly(vinyl alcohol), polyisoprene, etc. This broad applicability enabled a wide variety of applications, the list of which keeps growing (Figure 34A).^[^ ^13^ ^]^ The efficacy in crosslinking and the properties of the resulting materials depend heavily on the crosslinkers’ structure (e.g., flexibility of the linker) and physical properties (e.g., melting point or solubility). This explains the diversity of multivalent diazirines reported to date, a subset of which (**99**‐**102**) is displayed in Figure 34C.

Whereas the diazirine moieties carry the carbene‐precursor ability, the linker can also bear a given function. For instance, Wulff et al. designed a series of cleavable crosslinkers with embedded reactive groups (carbonate, oxalate, siloxane) that could be selectively triggered by an acid, base, and/or fluoride, allowing recycling and post‐functionalization of thermosets (Figure 34D).^[^ ^129^ ^]^ An analogous reasoning led Kumar, Rovis, and Chen to the design of universal *dynamic* crosslinkers by integrating dynamic covalent bonds in their linker (e.g., anhydride, thioester, or disulfide bonds).^[^ ^130^ ^]^ This allowed not only the compatibilization of immiscible mixed plastic waste but also the reprocessing of the resulting vitrimers. A similar approach based on dynamic imine covalent chemistry was reported by Li et al. a few months later.^[^ ^131^ ^]^

**Figure 35:**
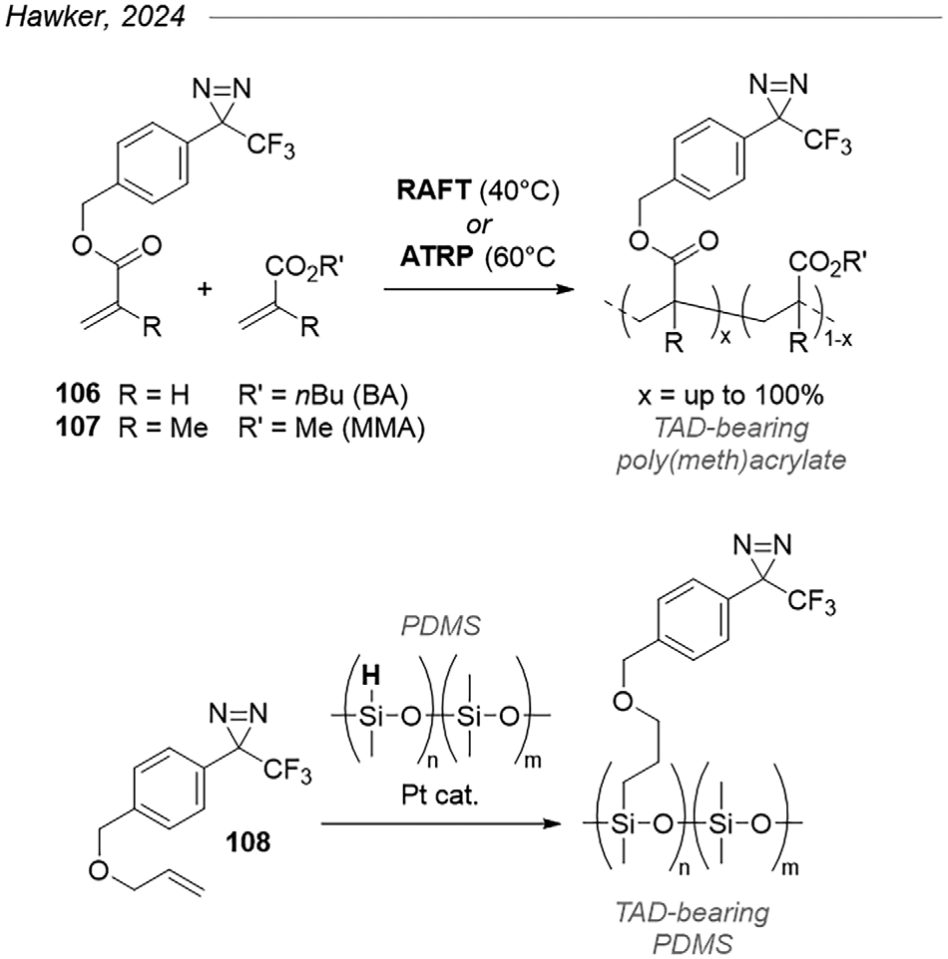
Synthesis of TAD‐bearing polymers via radical polymerization.

Hawker et al. pushed the concept of multivalent diazirine crosslinkers to its fullest by preparing TAD‐bearing polymers of controlled molecular weight by radical polymerization (Figure 35, top).^[^ ^132^ ^]^ To achieve this, they first synthesized the acrylate (**106**) and methacrylate (**107**) derivatives of the commercial *p*‐hydroxymethyl TPD and copolymerized them respectively with *n*‐butyl acrylate (*n*BA) or methyl methacrylate (MMA). They found that either RAFT (at 40 °C) or ATRP (at 60 °C) conditions were compatible with the diazirine moiety, which is worth noting considering the capacity of the N═N double bond to accept radicals (see above). The generally narrow molecular weight distributions of the obtained polymers (Ð < 1.5 for most examples) and their ^19^F NMR spectra suggest no premature crosslinking during radical polymerization. They were able to prepare poly(meth)acrylate copolymers bearing a tunable fraction of diazine‐bearing esters, as well as the homopolymer made of 100% of either **106** or **107**. RAFT copolymerization of **107** with styrene was also achieved in good yields (not shown here),^[^ ^132^ ^]^ and later the allyl ether analogue **108** was used to functionalize PDMS via Pt‐catalyzed hydrosilylation (Figure 35, bottom).^[^ ^133^ ^]^ The diazirine‐armed polymers could self‐crosslink under classical heat or UV activation or in a blend with unfunctionalized polymers.^[^ ^133^ ^]^ Diazirine loadings as low as 0.8 wt% were enough to obtain > 95% gel fraction, confirming the potential of multivalent diazirines in promoting rational crosslinking.

Given the inherent difficulty of characterizing crosslinked materials (due to their general insolubility), those reports presented a broad variety of tests to support their claims, thereby collecting valuable information regarding the properties and reactivity of diazirine‐born carbenes.

Although all these advances brought diazirines closer to industrial applications, the current synthetic costs imply that multivalent diazirine crosslinkers are best used so far in high‐value applications such as negative‐tone photopatterning in microelectronics^[^ ^134^ ^]^ (through crosslinking of low‐dielectric polymers like poly(HNB), which are otherwise difficult to crosslink) or as covalent adhesives (through crosslinking of surfaces).^[^ ^135^ ^]^ Additionally, the recent rise of concerns around PFAS in the environment prompts the design of fluorine‐free diazirine reagents for applications in materials science.

## 9 Conclusion and Perspectives

Six decades after their discovery, diazirines are just starting to be recognized for their utility outside of PAL. Their usage in organic synthesis certainly suffered from the shadow cast by diazo compounds, which were more readily synthesized and tamed. Therefore, many reports on diazirines before 2000 primarily studied the fundamental behavior of their carbenes and intermediates. Since then, streamlined synthetic access, more thorough characterizations, a more comprehensive understanding of their physicochemical properties, and, particularly, the advent of modern (and widely available) photochemical equipment triggered a renewed interest in this class of compounds. The past decade in particular has seen tremendous progress in synthetic applications, driven by skeletal editing and electrophilic amination strategies relying on chloro‐ and trifluoromethyl diazirines, respectively. While PAL concepts had slowly permeated toward (bio)materials science as early as the 1990s, the last decade witnessed the rise of diazirine reagents for universal functionalization and crosslinking of all sorts of materials.

The versatility of diazirines not only stems from their multiple applications but also from the diversity of substituents they accommodate and the many ways their intrinsic potential can be released. Indeed, their structure features a remarkably high heteroatom density and yields highly reactive species. Naturally, better control of their reactivity is desirable to achieve more accurate transformations, such as stereo‐ or regio‐selective reactions or chemoselective functionalization of (advanced) materials. Without any doubt, the development of diazirine chemistry will lead to yet unforeseeable strategies to assemble ever more complex molecules via the efficient building of new covalent bonds with these high‐energy reagents. In this latter aspect, diazirines are prime members in the select club of high‐energy dinitrogen‐bearing molecules, along with their not‐so‐distant cousins: diazoalkanes and diazocarbonyls, of course, but also diazonium salts, tetrazines, or even the lesser‐known diazetines,^[^ ^136^ ^]^ their four‐membered homologues. These energy‐packed, often small structures appear particularly relevant in a time when high reactivity must be achieved with atom economy and minimal waste, harmless nitrogen gas being the only by‐product of their reactions.

Most diazirines are stable in ambient conditions, can be used as off‐the‐shelf reagents, and activated on demand, releasing chemical energy along with the least concerning waste one can think of. In these regards, they should hold a privileged place in the toolbox of the modern synthetic chemist.

## Conflict of Interest

The authors declare no conflict of interest.

## Supporting information



Supporting Information

## Data Availability

Data sharing is not applicable to this article as no new data were created or analyzed in this study.
