# Supplementary material for: The Versatility of Diazirines: Properties, Synthetic and Modern Applications
Source: Chemistry. 2025 Jul 11;31(43):e202500414. doi: 10.1002/chem.202500414 (PMC12319365; doi:10.1002/chem.202500414)
Supplement: Supplementary file 1 — Supporting Information [file CHEM-31-e202500414-s001.pdf]

Supporting information

The Versatility of Diazirines: Properties, Synthetic and Modern Applications

Mathieu L. Lepage* and Emmanuel Gras*

NMR data (Figure 5)

Solvent is $CDCl_3$ unless specified otherwise. n.r. = not reported; n.o. = not observed.

Compound	Name	^{19}F (CF_3)	^{13}C (CF_3)	^{13}C (CCF_3)	^{1}H ($CHCF_3$)	Ref.
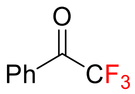	Trifluoro acetophenone	– 71 ppm	117 ppm (q, 291 Hz)	180 ppm (q, 35 Hz)	-	1
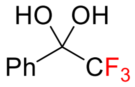	Trifluoro acetophenone hydrate	– 85 ppm	n.r.	n.r.	-	2
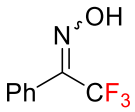	Trifluoro acetoxime	– 61 / – 66 ppm (measured by the authors of this review)	121 ppm (q, 275 Hz)	148 ppm (q, 33 Hz)	-	3
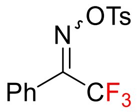	Tosylated trifluoro acetoxime	*major isomer* – 67 ppm<br>*minor isomer* – 61 ppm	*major isomer* 120 ppm (q, 278 Hz)<br>*minor isomer* 117 ppm (q, 285 Hz)	*major isomer* 154 ppm (q, 33 Hz)<br>*minor isomer* 154 ppm (q, 32 Hz)	-	4
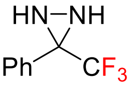	Diaziridine	– 76 ppm	124 ppm (q, 278 Hz)	58 ppm (q, 36 Hz)	-	4
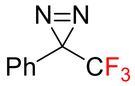	Diazirine	– 65 ppm	122 ppm (q, 275 Hz)	28 ppm (q, 40 Hz)	-	4
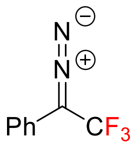	Diazoalkane<br>*(in C_6D_6)*	– 57 ppm<br>*in C_6D_6*	127 ppm (q, 269 Hz)<br>*in C_6D_6*	n.o.	-	5, 6
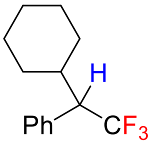	C–H insertion product in cyclohexane:<br>(1-cyclohexyl-2,2,2-trifluoroethyl)benzene	– 63 ppm (d, 10.2 Hz)	127 ppm (q, 281 Hz)	56 ppm (q, 25 Hz)	3.03 ppm (qd, 10.2, 8.0 Hz)	7, 8

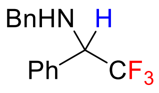	N–H insertion product in benzylamine:<br><br>*N*-benzyl-2,2,2-trifluoro-1-phenylethan-1-amine	– 74 ppm (d, 7.3 Hz)	125 ppm (q, 281 Hz)	64 ppm (q, 29 Hz)	4.22 ppm (q, 7.3 Hz)	9
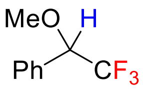	O–H insertion product in methanol:<br><br>(2,2,2-trifluoro-1-methoxyethyl)benzene	– 77 ppm (d, 6.6 Hz)	124 ppm (q, 282 Hz)	82 ppm (q, 31 Hz)	4.50 ppm (q, 6.6 Hz)	10
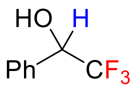	O–H insertion product in water:<br><br>2,2,2-trifluoro-1-phenylethan-1-ol	– 79 ppm (d, 6.7 Hz)	124 ppm (q, 282 Hz)	73 ppm (q, 32 Hz)	5.00 ppm (q, 6.7 Hz)	11, 12
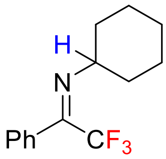	Cyclohexyl imine	– 71 ppm	120 ppm (q, 280 Hz)	156 ppm (q, 33 Hz)	$C\mathbf{H}N=CCF_3$<br><br>3.23 ppm (m)	13
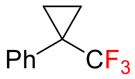	Cyclopropane	– 73 ppm	127 ppm (q, 274 Hz)	29 ppm (q, 34 Hz)	-	14
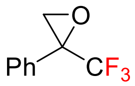	Epoxide	– 78 ppm	123 ppm (q, 276 Hz)	58 ppm (q, 38 Hz)	-	15
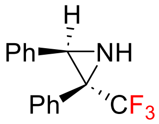	Aziridine	– 73 ppm	129 ppm (q, 279 Hz)	48 ppm (q, 33 Hz)	-	16
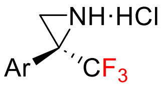	Aziridinium chloride (Ar = 4-MeOPh)	– 76 ppm	126 ppm (q, 287 Hz)	62 ppm (q, 26 Hz)	-	17
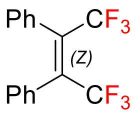	(*Z*)-stilbene product of carbene dimerization	– 58 ppm	122 ppm (q, 275 Hz)	138 ppm (m)	-	18
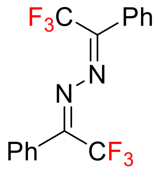	Azine product	– 67 ppm<br><br>(+11.5 ppm vs. TFA)	120 ppm (q, 277 Hz)	144 ppm (q, 35 Hz)	-	19

Electrochemical data (Figure 11)

For the purpose of clarity, the information depicted in **Figure 11** has been simplified and electric potentials have been approximated. Below are compiled the data as described in the original articles.

Elson, C. M.; Liu, M. T. H. Electrochemical behaviour of diazirines. *J. Chem. Soc., Chem. Commun.* **1982**, 415-416. DOI: 10.1039/C39820000415.

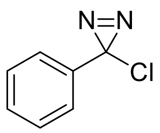	*E*p = – 1.87 V at a vitreous carbon electrode "relative to an Ag / $AgNO_3$ (0.1 M in MeCN) reference electrode"<br>Tetraethylammonium perchlorate ($Et_4N{\cdot}ClO_4$) was used as electrolyte.<br>Irreversible reduction.
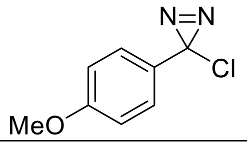	*E*p = – 2.05 V<br>Same behavior as phenyl chloro diazirine but with a cathodic shift of – 0.19 V.
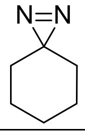	No reduction wave found between 0 and – 2.7 V
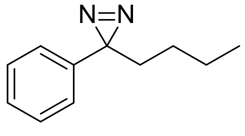	*E*p = – 2.47 V<br>"…difference in cathodic peak potentials between phenyl chloro diazirine and butyl phenyl diazirine was approximately – 0.6 V."<br>Reversible reduction.

Elson, C. M.; Liu, M. T. H.; Mailer, C. E.s.r. studies of diazirine anion radicals. *J. Chem. Soc., Chem. Commun.* **1986**, *7*, 504-506, 10.1039/C39860000504. DOI: 10.1039/C39860000504.

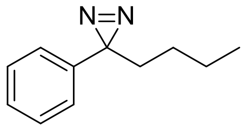	One-electron reversible reduction at – 2.55 V (Ep_c) in MeCN–Et_4NClO_4 (0.1 M).<br>$t_{1/2}$ = 13 s at 10°C
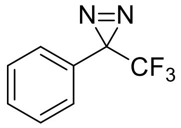	Similar behavior at – 1.94 V (Ep_c).<br>$t_{1/2}$ = 46 s at 10°C

Lawrence, E. J.; Wildgoose, G. G.; Aldous, L.; Wu, Y. A.; Warner, J. H.; Compton, R. G.; McNaughter, P. D. 3-Aryl-3-(trifluoromethyl)diazirines as Versatile Photoactivated "Linker" Molecules for the Improved Covalent Modification of Graphitic and Carbon Nanotube Surfaces. *Chem. Mater.* **2011**, *23* (16), 3740-3751. DOI: 10.1021/cm201461w.

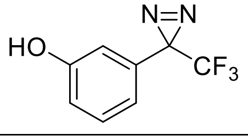	E (V) at vitreous carbon electrode: – 1.86 V vs. ferrocene (1 mM in MeCN + 0.1 M Bu_4NBF_4).<br>Scan rate = 100 mV/s → irreversible reduction<br>Scan rate > 900 mV/s → partially reversible
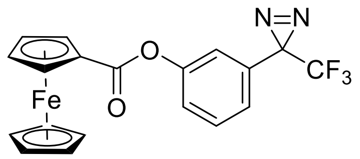	E (V) at vitreous carbon electrode: – 1.83 V vs. ferrocene (1 mM in MeCN + 0.1 M Bu_4NBF_4).

Ping, J.; Gao, F.; Chen, J. L.; Webster, R. D.; Steele, T. W. J. Adhesive curing through low-voltage activation. *Nat. Commun.* **2015**, *6* (1), 8050. DOI: 10.1038/ncomms9050.

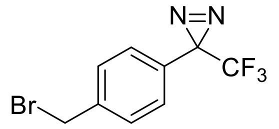	E (V) at glassy carbon electrode: – 1.6 V vs. Ag / AgCl (1 mM in MeCN + 0.1 M Et_4NBF_4).<br>Scan rate = 100 mV/s → partially reversible reduction<br>Scan rate = 1 V/s → fully reversible

References for the supporting information

(1) Wu, W.; Tian, Q.; Chen, T.; Weng, Z. Copper-Mediated Trifluoroacetylation of Arenediazonium Salts with Ethyl Trifluoropyruvate. *Chem. Eur. J.* **2016**, *22* (46), 16455-16458. DOI: 10.1002/chem.201604300.
(2) Jiang, X.-D.; Matsukawa, S.; Kakuda, K.-I.; Fukuzaki, Y.; Zhao, W.-L.; Li, L.-S.; Shen, H.-B.; Kojima, S.; Yamamoto, Y. Efficient synthesis of tetradecafluoro-4-phenylheptan-4-ol by a Cannizzaro-type reaction and application of the alcohol as a bulky Martin ligand variant for a new anti-apicophilic phosphorane. *Dalton Trans.* **2010**, *39* (41), 9823. DOI: 10.1039/c0dt00539h.
(3) Protasova, I.; Bulat, B.; Jung, N.; Bräse, S. Synthesis of Diaziridines and Diazirines via Resin-Bound Sulfonyl Oximes. *Org. Lett.* **2017**, *19* (1), 34-37. DOI: 10.1021/acs.orglett.6b03252.
(4) Tanbouza, N.; Carreras, V.; Ollevier, T. Photochemical Cyclopropenation of Alkynes with Diazirines as Carbene Precursors in Continuous Flow. *Org. Lett.* **2021**, *23* (14), 5420-5424. DOI: 10.1021/acs.orglett.1c01750.
(5) Hyde, S.; Veliks, J.; Ascough, D. M. H.; Szpera, R.; Paton, R. S.; Gouverneur, V. Enantioselective rhodium-catalysed insertion of trifluorodiazoethanes into tin hydrides. *Tetrahedron* **2019**, *75* (1), 17-25. DOI: 10.1016/j.tet.2018.11.022.
(6) Emer, E.; Twilton, J.; Tredwell, M.; Calderwood, S.; Collier, T. L.; Liégault, B.; Taillefer, M.; Gouverneur, V. Diversity-Oriented Approach to CF3CHF-, CF3CFBr-, CF3CF2-, (CF3)2CH-, and CF3(SCF3)CH-Substituted Arenes from 1-(Diazo-2,2,2-trifluoroethyl)arenes. *Org. Lett.* **2014**, *16* (22), 6004-6007. DOI: 10.1021/ol5030184.
(7) Musolino, S. F.; Pei, Z.; Bi, L.; DiLabio, G. A.; Wulff, J. E. Structure–function relationships in aryl diazirines reveal optimal design features to maximize C–H insertion. *Chem. Sci.* **2021**, *12* (36), 12138-12148. DOI: 10.1039/D1SC03631A.
(8) Liu, Z.; Cao, S.; Yu, W.; Wu, J.; Yi, F.; Anderson, E. A.; Bi, X. Site-Selective C–H Benzylation of Alkanes with N-Triftosylhydrazones Leading to Alkyl Aromatics. *Chem* **2020**, *6* (8), 2110-2124. DOI: 10.1016/j.chempr.2020.06.031.
(9) Chen, H.; Ye, J.-L.; Huang, P.-Q. Chemoselective direct reductive trifluoromethylation of amides: a flexible access to functionalized α-trifluoromethylamines. *Org. Chem. Front.* **2018**, *5* (6), 943-947. DOI: 10.1039/c7qo01031a.
(10) Cabanero, D. C.; Kariofillis, S. K.; Johns, A. C.; Kim, J.; Ni, J.; Park, S.; Parker, D. L.; Ramil, C. P.; Roy, X.; Shah, N. H.; et al. Photocatalytic Activation of Aryl(trifluoromethyl) Diazos to Carbenes for High-Resolution Protein Labeling with Red Light. *J. Am. Chem. Soc.* **2024**, *146* (2), 1337-1345. DOI: 10.1021/jacs.3c09545.
(11) Prakash, G. K. S.; Zhang, Z.; Wang, F.; Munoz, S.; Olah, G. A. Nucleophilic Trifluoromethylation of Carbonyl Compounds: Trifluoroacetaldehyde Hydrate as a Trifluoromethyl Source. *J. Org. Chem.* **2013**, *78* (7), 3300-3305. DOI: 10.1021/jo400202w.
(12) Krishnamurti, R.; Bellew, D. R.; Prakash, G. K. S. Preparation of trifluoromethyl and other perfluoroalkyl compounds with (perfluoroalkyl)trimethylsilanes. *J. Org. Chem.* **1991**, *56* (3), 984-989. DOI: 10.1021/jo00003a017.
(13) Shu, X.; Xu, R.; Liao, S. Photocatalytic divergent decarboxylative amination: a metal-free access to aliphatic amines and hydrazines. *Sci. China Chem.* **2021**, *64* (10), 1756-1762. DOI: 10.1007/s11426-021-1048-4.

(14) Mercadante, M. A.; Kelly, C. B.; Hamlin, T. A.; Delle Chiaie, K. R.; Drago, M. D.; Duffy, K. K.; Dumas, M. T.; Fager, D. C.; Glod, B. L. C.; Hansen, K. E.; et al. 1,3-γ-Silyl-elimination in electron-deficient cationic systems. *Chem. Sci.* **2014**, *5* (10), 3983. DOI: 10.1039/c4sc01732c.
(15) Kavanagh, S. A.; Piccinini, A.; Connon, S. J. Efficient Catalytic Corey–Chaykovsky Reactions Involving Ketone Substrates. *Adv. Synth. Catal.* **2010**, *352* (11-12), 2089-2093. DOI: 10.1002/adsc.201000255.
(16) Félix, C. P.; Khatimi, N.; Laurent, A. J. Stereoselective addition of CF3SiMe3 on azirines. Synthesis of (E)-aziridines. *Tetrahedron Lett.* **1994**, *35* (20), 3303-3304. DOI: 10.1016/S0040-4039(00)76891-2.
(17) Yang, Y.; Huang, Y.; Qing, F.-L. Asymmetric synthesis of trifluoromethylated aziridines from CF_3-substituted *N-tert*-butanesulfinyl ketimines. *Tetrahedron Lett.* **2013**, *54* (29), 3826-3830. DOI: 10.1016/j.tetlet.2013.05.048.
(18) Zhao, B.; Li, Y.; Tu, D.-H.; Zhang, W.; Liu, Z.-T.; Lu, J. Palladium catalyzed mono and difunctionalization of hexafluorobut-2-yne. *Tetrahedron Lett.* **2016**, *57* (39), 4345-4347. DOI: 10.1016/j.tetlet.2016.08.025.
(19) Benomar, S.; Patel, B.; E. Tipping, A. Unsaturated nitrogen compounds containing fluorine. Part 7[1]. The reaction of 2,5-dichloro-1,1,1,6,6,6-hexafluoro-3,4-diazahexa-2,4-diene with phenyl-lithium. *J. Fluorine Chem.* **1990**, *50* (2), 207-215. DOI: 10.1016/S0022-1139(00)80496-0.
